# Molecular evolution of the Wood-Ljungdahl pathway and the reductive glycine pathway in *Desulfobacterota*

**DOI:** 10.3389/fmicb.2025.1708584

**Published:** 2026-01-09

**Authors:** Tomoyuki Wakashima, Keitaro Kume, Yoko Chiba

**Affiliations:** 1 RIKEN Center for Sustainable Resource Science, Saitama, Japan; 2 Graduate School of Science and Technology, University of Tsukuba, Ibaraki, Japan; 3 Institute of Medicine, University of Tsukuba, Ibaraki, Japan; 4 Center for Cyber Medicine Research, University of Tsukuba, Ibaraki, Japan; 5 Institute of Life and Environmental Sciences, University of Tsukuba, Ibaraki, Japan

**Keywords:** bacterial evolution, carbon fixation, carbon metabolism, molecular evolution, reductive acetyl-CoA pathway, reductive glycine pathway, *Thermodesulfobacteriota*

## Abstract

Carbon fixation is a fundamental metabolic process that sustains ecosystems, yet its origins and evolutionary history remain largely unresolved. In this study, we focused on the Wood-Ljungdahl (WL) pathway, which is considered one of the most ancient carbon fixation pathways, and the reductive glycine (rGly) pathway, which shares several reactions with the WL pathway. The evolutionary scenario of the two carbon fixation pathways was inferred in the phylum *Desulfobacterota*, which includes microorganisms that operate either the WL pathway or the rGly pathway for autotrophic growth. The timing of gene gain and loss events was inferred by gene presence/absence analyses for both pathways, together with phylogenetic analyses of their key enzymes. Our results suggested that the common ancestor of *Desulfobacterota* possessed all genes encoding key enzymes of both pathways; formate dehydrogenase, the carbon monoxide dehydrogenase/acetyl-CoA synthase complex and the glycine cleavage system. Furthermore, analyses of complete gene sets for the WL and rGly pathways, together with downstream genes required for amino acid biosynthesis, supported the possibility that the common ancestor of this phylum had been capable of autotrophic growth through these carbon fixation pathways. Then, multiple lineages have lost the WL and rGly pathway genes independently during subsequent evolution. Gene replacements also occurred in the glycine cleavage system by regaining genes by horizontal gene transfer. These results suggest that carbon fixation pathways in extant organisms in the phylum *Desulfobacterota* arose through a combination of vertical inheritance, gene loss, and horizontal gene transfer.

## 1 Introduction

Elucidating the origins and early evolution of carbon fixation pathways is essential to understand the origin of life. Whether the first organisms on Earth were autotrophs or heterotrophs remains unresolved (Wächtershäuser, 1988; Bada and Lazcano, 2002; Kitadai and Maruyama, 2018; Kitadai et al., 2021), however, there is no doubt that the emergence of autotrophs was crucial for establishing ecosystems on Earth. This is because organic compounds produced solely through abiotic processes are insufficient to sustain a substantial biomass (Martin and Russell, 2007; Berg et al., 2010; Weiss et al., 2016; Nunoura et al., 2018; Dick, 2019). There is growing interest in how early life fixed carbon dioxide (CO_2_) and how it evolved to acquire the complex and highly refined metabolic systems observed today (Fuchs, 2011; Braakman and Smith, 2012; Moody et al., 2024).

To date, seven carbon fixation pathways have been identified in extant organisms in nature (Berg et al., 2010; Fuchs, 2011; Sánchez-Andrea et al., 2020). Among these pathways, the Wood-Ljungdahl (WL) pathway, which is also called the reductive acetyl-CoA pathway, is considered to be one of the oldest ones and existed in the last universal common ancestor (LUCA), owing to its broad conservation across diverse lineages including bacteria and archaea (Peretó et al., 1999; Martin and Russell, 2007; Berg et al., 2010; Braakman and Smith, 2012; Nitschke and Russell, 2013; Schuchmann and Müller, 2014; Weiss et al., 2016; Moody et al., 2024). The old origin of the WL pathway is also supported by its structural simplicity and high energy efficiency (Martin and Russell, 2007; Berg et al., 2010; Fuchs, 2011; Schuchmann and Müller, 2014).

**Figure 1:**
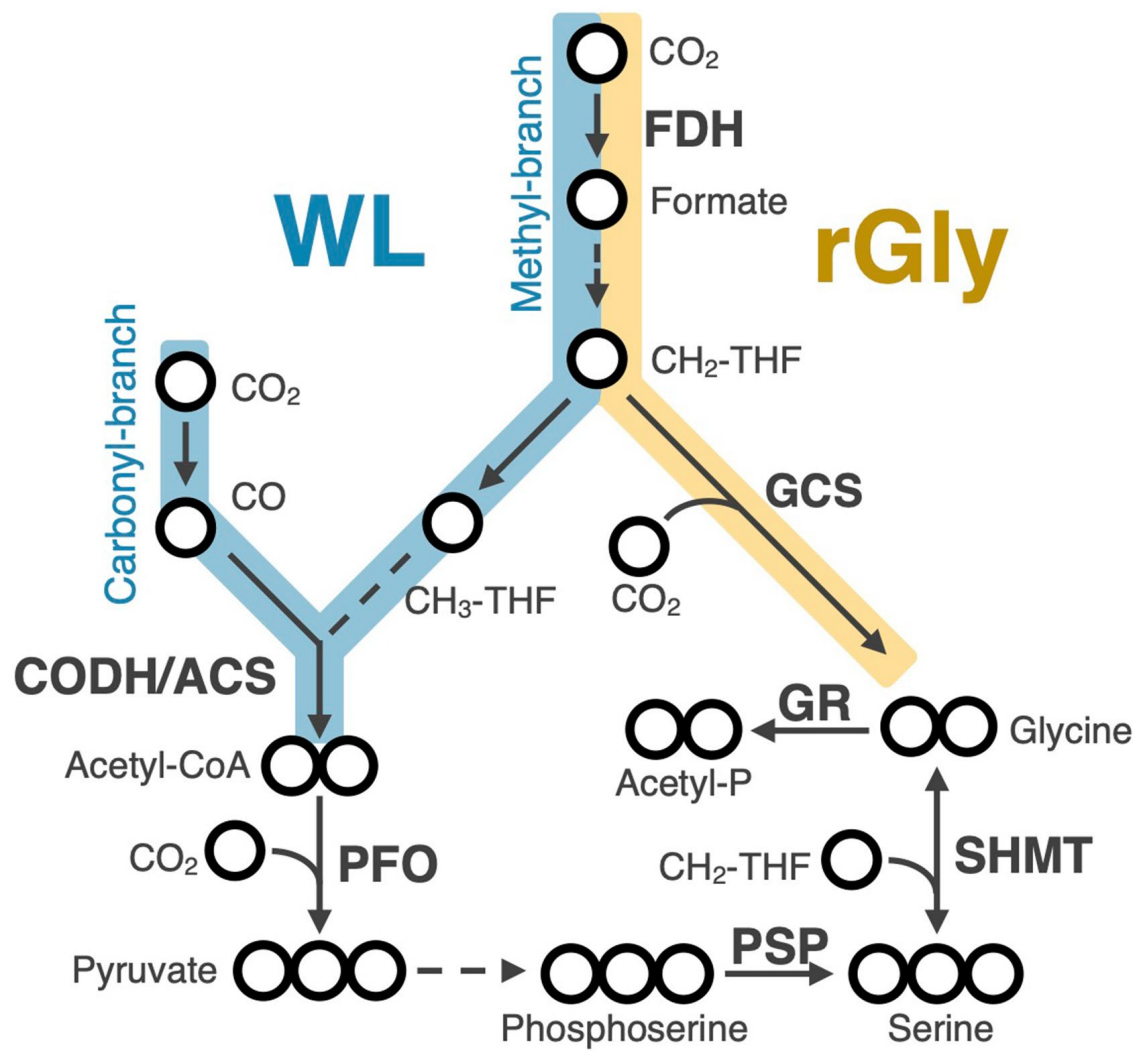
Metabolic map of the Wood-Ljungdahl (WL) and reductive glycine (rGly) pathways. Blue and yellow lines indicate reactions in the WL and rGly pathways, respectively. Arrows represent enzymatic reactions and dashed arrows indicate reactions catalyzed by multiple enzymes. Bold letters show enzyme names. Circles represent number of carbons. THF, Tetrahydrofolate; FDH, formate dehydrogenase; CODH/ACS, carbon monoxide dehydrogenase/acetyl-CoA synthase; GCS, glycine cleavage system; PFO, pyruvate-ferredoxin oxidoreductase; SHMT, serine hydroxymethyltransferase; PSP, phosphoserine phosphatase; GR, glycine reductase.

The WL pathway incorporates two molecules of CO_2_ through the methyl and the carbonyl branches to synthesize one molecule of acetyl-CoA (Figure 1, highlighted in blue) (Schuchmann and Müller, 2014). In the methyl branch of the bacterial WL pathway, CO_2_ is converted to formate by formate dehydrogenase (FDH) and subsequently reduced to a methyl group (-CH_3_) bound to tetrahydrofolate (THF) to produce CH_3_-THF. In the carbonyl branch, CO_2_ is reduced to CO by carbon monoxide dehydrogenase (CODH) and then combines with the methyl group of the CH_3_-THF and CoA to become acetyl-CoA by acetyl-CoA synthase (ACS). CODH and ACS involved in the WL pathway form a complex composed of five subunits; AcsA, AcsB, AcsC, and AcsD, are conserved between bacteria and archaea and AcsE and CdhB are specific to bacteria and archaea, respectively (Supplementary Table 1). In both bacteria and archaea, the genes for these five subunits form a cluster (Adam et al., 2018). By contrast, a homolog of AcsA (CooS) is often encoded independently outside the CODH/ACS gene cluster and does not function in the WL pathway (Techtmann et al., 2012; Adam et al., 2018; Inoue et al., 2019). Both genetic and biochemical studies have confirmed that this pathway actually functions for carbon fixation in a wide range of taxa, including methanogens, acetogens, and sulfate-reducing bacteria, spanning both archaeal and bacterial domains (Schuchmann and Müller, 2014; Adam et al., 2018; Borrel et al., 2016; Pierce et al., 2008).

The reductive glycine (rGly) pathway (Figure 1, highlighted in yellow) is the most recently identified CO_2_ fixation pathway that supports the autotrophic growth of organisms in nature (Sánchez-Andrea et al., 2020; Song et al., 2020). This pathway utilizes the reverse reaction of the glycine cleavage system (GCS), which cleaves glycine to methylene tetrahydrofolate (CH_2_-THF), CO_2_, and ammonia (Figure 1, highlighted in yellow) (Kikuchi, 1973; Kikuchi et al., 2008; Kim et al., 2017). GCS is composed of five proteins named the T-protein, P-protein *α* subunit, P-protein *β* subunit, L-protein, and H-protein (GCST, GCSPα, GCSPβ, GCSL, and GCSH, respectively), and their genes are known to form a cluster (Okamura-Ikeda et al., 1993; Kikuchi et al., 2008; Lorio et al., 2010; Tezuka and Ohnishi, 2014; Cao et al., 2018). GCSP catalyzes the decarboxylation of glycine and the product, aminomethyl group (-CH_2_-NH_2_), is transferred to a carrier protein, GCSH. The aminomethyl group reacts with tetrahydrofolate (THF) to produce CH_2_-THF and ammonia, which is catalyzed by GCST. The released GCSH is then re-oxidized by GCSL, a FAD-dependent oxidoreductase (Kikuchi et al., 2008; Kikuchi, 1973). Because the GCS reaction is thermodynamically near equilibrium (Kikuchi et al., 2008), glycine synthesis through the reverse GCS reaction to supplement organic compounds has been known for a long time in some heterotrophs (Waber and Wood, 1979; Dürre and Andreesen, 1982; Fuchs, 1986; Schneeberger, 1999). On the other hand, biochemically demonstrated CO_2_ fixation to support the autotrophic growth via the reverse GCS reaction (i.e., the rGly pathway) is limited to two species; *Desulfovibrio desulfuricans* belonging to the phylum *Desulfobacterota* (Sánchez-Andrea et al., 2020) and *Clostridium drakei* belonging to the phylum *Bacillota* (Song et al., 2020). Notably, the pathway to provide CH_2_-THF from CO_2_ is shared between the rGly and WL pathways (Figure 1; Supplementary Figure 1) (Sánchez-Andrea et al., 2020), suggesting profound functional and evolutionary relationships between them (Cotton et al., 2018; Song et al., 2020). In fact, *Clostridium drakei* has been biochemically proven to operate both the rGly and WL pathways for CO_2_ fixation (Song et al., 2020). Furthermore, the last bacterial common ancestor (LBCA) is suggested to have retained some part of the genes for these metabolic pathways. Ancestral metabolic reconstruction based on the gene phylogenies across major bacterial lineages identified methyl-branch enzyme genes shared between the WL and rGly pathways in the LBCA, although it did not support the presence of FDH, which catalyzes the first reaction in these pathways (Coleman et al., 2021).

FDH working for the WL or rGly pathways forms a heteromeric protein complex (Maia et al., 2015). For example, the FDH in *Moorella thermoacetica*, a model bacterium for the WL pathway, has an *α*_2_*β*_2_ structure (Yamamoto et al., 1983). In contrast, the FDH in *D. desulfuricans*, which operates the rGly pathway, consists of three subunits; α, β, and *γ* (Maia et al., 2016). The FDHα subunit in both species contains a metal that binds to CO_2_ and belongs to the MopB superfamily, however, they are categorized into distinct groups; the FDHα subunits of *M. thermoacetica* and *D. desulfuricans* are categorized into cytoplasmic FDH and FdhG, respectively, based on the distinct clade on their phylogenetic tree (Wells et al., 2023). In contrast, FDHβ exhibits no homology between in *M. thermoacetica* and *D. desulfuricans* and is therefore expected to have different evolutionary origin. The phylogenetic diversity of FDH makes it difficult to estimate the existence/absence of the gene in ancestral organisms based on phylogenetic analysis, because the estimation is influenced by the query, taxon sampling, and root positions (Superson et al., 2019; Coleman et al., 2021; Powell and Battistuzzi, 2022).

Here, we estimated the evolutionary scenario of the genes related to CO_2_ fixation pathways in a bacterial phylum *Desulfobacterota* (Waite et al., 2020). The phylum *Desulfobacterota* belongs to the *Gracilicutes* within the bacterial domain (Coleman et al., 2021) and is phylogenetically related to the phyla *Myxococcota*, *Bdellovibrionota*, and *Proteobacteria* (Supplementary Figure 2). Waite et al. reclassified the proteobacterial classes and proposed a new phylum of *Desulfobacterota* that consists of organisms previously classified in the class *Deltaproteobacteria* and in the phylum *Thermodesulfobacteria*.

The phylum *Desulfobacterota* includes numerous thermophilic sulfate-reducing organisms from hydrothermal vent environments (Moussard et al., 2004; Alain et al., 2010; Frolova et al., 2018), which are widely regarded as candidate sites for the origin and early evolution of life (Martin and Russell, 2007; Nitschke and Russell, 2013; Dick, 2019). Furthermore, the phylum *Desulfobacterota* includes several chemolithoautotrophic bacteria that operate either the rGly or WL pathway. For example, *Desulfovibrio desulfuricans* fixes CO_2_ via the rGly pathway (Sánchez-Andrea et al., 2020), while *Thermodesulfatator indicus*, *Dissulfurirhabdus thermomarina*, and *Desulfobacterium autotrophicum* utilize the WL pathway for autotrophic growth (Brysch et al., 1987; Allioux et al., 2020; Chiba et al., 2025). Therefore, this phylum is expected to be a relevant model to estimate the presence or absence of the gene set for the rGly and WL pathways in ancestral organisms and how it evolved to the extant organisms which have either pathway. To access these questions, we conducted presence/absence analyses for both pathway genes to identify the gene sets in extant organisms and performed phylogenetic analyses to estimate how the genes have been inherited.

## 2 Materials and methods

### 2.1 Gene presence/absence analysis

To examine the gene presence/absence, we selected at least one representative species from each of the 42 families within the phylum *Desulfobacterota* based on the classification proposed by Waite et al. (2020). Priority was given to those whose genomes are registered in RefSeq. To minimize false negatives in presence/absence calls due to incomplete assemblies, we restricted our analyses to RefSeq genomes that are annotated as complete genome and designated as reference or representative genomes by NCBI. These assemblies have passed the RefSeq curation and quality control pipeline (O’Leary et al., 2016; Li et al., 2021). As an exception, for uncultured lineages (Candidatus *Magnetomorum* sp., Candidatus *Adiutrix intracellularis*, *Smithella* sp., *Trichloromonas* sp., GWC2_55_46 and Dadabacteria bacterium) that lack complete genomes in RefSeq, we used the metagenome-assembled genomes (MAGs). For all the selected species, we checked their culture conditions based on the literature. Species that have been experimentally confirmed to grow using CO_2_ as the sole carbon source were determined as autotrophs (Supplementary Table 2).

Proteomes for each representative species were predicted from NCBI RefSeq, and the presence or absence of enzymes involved in the WL and rGly, and subsequent amino acid synthesis pathways was estimated by homology searches using BLAST (Altschul et al., 1990). As queries, we used 39 amino acid sequences of the enzymes from *M. thermoacetica, D. desulfuricans, T. indicus, Escherichia coli, Thermus thermophilus* and *Hydrogenobacter thermophilus* (Supplementary Table 3). In the BLAST searches, we defined the homologs as those with an e-value of <1e-5 and query coverage of 70% or more.

In this study, we defined the FDHα subunit, the CODH/ACS complex, and the GCS complex as the key enzymes of the WL and rGly pathways. We inferred that a species possessed the WL pathway if its genome encoded both a homolog of FDHα subunit and all five proteins of the CODH/ACS complex; AcsA, AcsB, AcsC, AcsD, and AcsE. We inferred that a species possessed the rGly pathway if its genome encoded homologs of FDHα and all the five GCS complex proteins; GCSPα, GCSPβ, GCST, GCSL, and GCSH.

### 2.2 Phylogenetic analyses

The target enzymes for phylogenetic analyses are as follows: FDH, the CODH/ACS complex, the GCS complex, pyruvate-ferredoxin oxidoreductase (PFO), phosphoserine phosphatase (PSP), serine hydroxymethyltransferase (SHMT), and the glycine reductase (GR) complex. Finally, a total of 28 enzyme subunits were the subjects of the analyses (Supplementary Table 4).

We collected sequences by MMseqs2 (release 14-7e284) similarity searches (Steinegger and Söding, 2017) against a local NCBI-nr database (ver. 2024/Nov/03). In these searches, we used sequences whose enzymatic functions were biochemically analyzed (Supplementary Table 4). The command line options of MMseqs2 search were “-e 1e-5 -s 7.0 --num-iterations 5.” From the result of the similarity search of each query, we first removed bottom 90% of MAGs-derived hits by e-value. Then, we selected one representative sequence from each of NCBI taxid set we specified to cover the diversity of the tree of life. This set includes 199 NCBI taxids: 139 from all the three domains of life and 60 from *Desulfobacterota* and closely related lineages (Supplementary Table 5). These representatives on each query were finally merged. Next, the domain structures of the merged sequences were identified using InterProScan ver. 5.72, and sequences whose domain structures did not match those of the queries were removed (Jones et al., 2014). Sequences with large insertions or deletions were also removed to ensure accurate alignments. Next, in the preliminary phylogeny, we manually excluded sequences that exhibited long branches (>1.5 substitution per site) in the preliminary phylogenetic trees to minimize the effects of long-branch attraction (LBA) artefact (Bergsten, 2005; Graybeal, 1998; Hedtke et al., 2006). Because more stringent filtering criteria were applied in this phylogenetic analysis compared to the preceding gene presence/absence analysis to avoid the LBA artefact, some sequences that had been identified as “present” in the preceding analysis were excluded from the phylogenetic analysis. The numbers of sequences ultimately used and excluded are listed in Supplementary Table 4.

The curated sequences were aligned using MUSCLE (Edgar, 2004) and subsequently trimmed with G-blocks (Talavera and Castresana, 2007). The following three options were enabled for trimming: I. Allow smaller final blocks, II. Allow gap positions within the final blocks, and III. Allow less strict flanking positions. Phylogenetic trees were then inferred by maximum likelihood method using IQ-TREE ver. 2.3.6 on the final trimmed alignment data (Minh et al., 2020). Model selection was performed using ModelFinder “-m MFP” (Kalyaanamoorthy et al., 2017), which automatically determined the best-fit model for each enzyme. Node support values were evaluated using ultrafast bootstrap analysis “-B 1000” (Hoang et al., 2018). The evolutionary models and the number of selected amino acid sites used to infer each phylogenetic tree were provided in the legends of the respective figures. The workflow for the phylogenetic analysis is illustrated in Supplementary Figure 10.

For FDHα, a single phylogenetic tree was inferred by combining the newly collected homologous sequences of W-FDH (cytoplasmic Fdhs) from *M. thermoacetica* and Mo-FDH (FdhG) from *D. desulfuricans* with known sequences from the MopB superfamily (Wells et al., 2023). Subsequently, these sequences were divided into cytoplasmic Fdhs and FdhG groups, and separate phylogenetic trees were inferred for each.

### 2.3 Genome synteny analysis of CODH/ACS complex

To determine whether the CODH/ACS functions as a complex involved in the WL pathway, we analyzed the genome synteny of the subunit genes for CODH/ACS. For all sequences used in the phylogenetic analysis of AcsA, we manually examined the genomic structures to verify whether all the AcsA used in the single-gene phylogenetic analysis formed a cluster with other subunits (AcsB to E).

## 3 Results

### 3.1 Presence or absence of carbon fixation pathway genes

**Figure 2:**
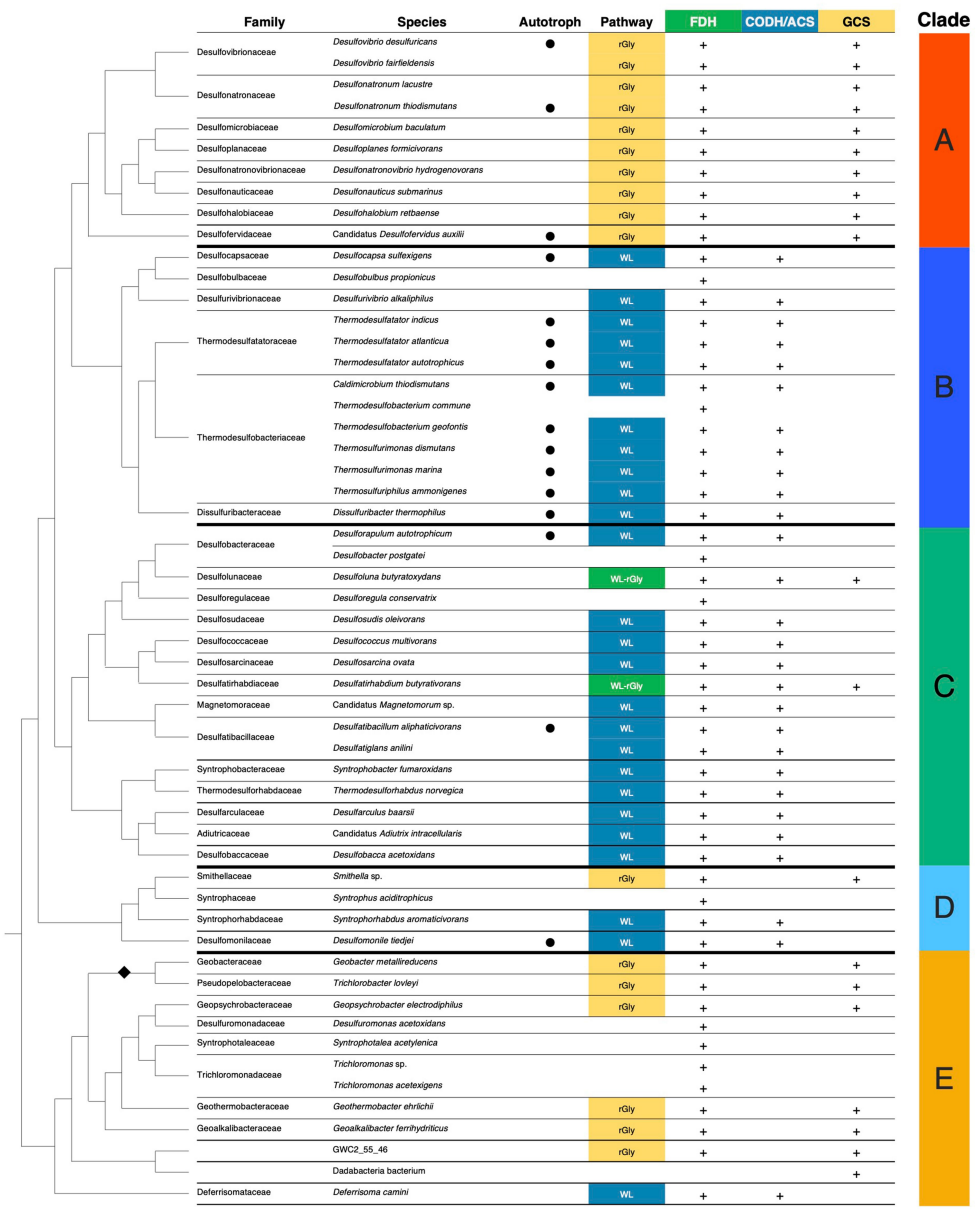
Presence or absence of enzymes for the WL and rGly pathways in *Desulfobacterota* species. The tree on the left shows a phylogenetic tree of *Desulfobacterota* species, modified from Waite et al. (2020). The order Geobacterales in clade E is indicated by a black diamond (◆). Autotrophic species are indicated by black circles (●) based on cultivation data from original species descriptions and/or subsequent studies. The presence or absence of each enzyme is based on predicted proteomes. FDH is considered to be present (+) when a species encodes either the homolog of cytoplasmic Fdh or FdhG. CODH/ACS is considered to be present if all five subunits of the CODH/ACS complex are encoded. GCS is considered present when all five proteins of the glycine cleavage system are encoded. Pathway: When CODH/ACS and GCS are present together with FDH, the species is estimated to have the WL and rGly pathways, respectively. Clade: The phylum *Desulfobacterota* was divided into five monophyletic clades in this study.

The presence or absence of all the 39 genes for enzymes involved in the WL and rGly pathways and subsequent amino acid synthesis was determined in 55 representative species in the phylum *Desulfobacterota*. Here, we classified the phylum *Desulfobacterota* into clade A to E (Figure 2; Supplementary Table 6) based on phylogenetic relationships and physiological characteristics such as habitat and optimal growth temperature. Literature search revealed at least 16 out of the 55 species can grow chemolithoautotrophically and they were distributed across clades A, B, C and D (Supplementary Table 2) (Brysch et al., 1987; DeWeerd et al., 1990; Finster et al., 1998; Pikuta et al., 2003; Cravo-Laureau et al., 2004; Moussard et al., 2004; Alain et al., 2010; Slobodkin et al., 2012; Hamilton-Brehm et al., 2013; Slobodkin et al., 2013; Kojima et al., 2016; Krukenberg et al., 2016; Lai et al., 2016; Mardanov et al., 2016; Slobodkina et al., 2017; Frolova et al., 2018; Sánchez-Andrea et al., 2020). In the 55 representative species, 50 and 44 species possessed the homologs of cytoplasmic Fdhs in *M. thermoacetica* and FdhG in *D. desulfuricans*, respectively (Supplementary Table 6C). All the analyzed species were found to have at least one FDHα candidate gene with only one exception of an uncultured *Candidatus* Dadabacteria bacterium (Figure 2). Furthermore, 41 species possessed both cytoplasmic Fdhs and FdhG homologs and the 41 species were distributed in all five clades (A-E) of the phylum *Desulfobacterota*. This suggests that the common ancestor of the phylum *Desulfobacterota* already possessed genes for both cytoplasmic Fdhs and FdhG.

The complete gene set for the CODH/ACS complex was identified in 27 species belonging to clades B, C, D, and E, including *T. indicus*, *D. thermomarina*, and *D. autotrophicum* which actually operate the WL pathway for CO_2_ fixation (Figure 2). The complete gene set for the GCS complex was identified in 21 species in clades A, C, D, and E including *D. desulfuricans*, which possesses the rGly pathway and belongs to clade A (Figure 2). Except for the uncultured *Candidatus* Dadabacteria bacterium, the organisms possessing the gene set for either the CODH/ACS or GCS complex also had FDH genes, suggesting that they may possess the WL or rGly pathway. In particular, species reported to grow autotrophically have high possibility of fixing CO_2_ via either the WL or rGly pathway. All the ten species analyzed in clade A had the gene set for the rGly pathway, whereas none of them had the WL pathway gene set. Conversely, in clade B, 11 out of 13 species conserved the WL pathway gene set, and no species were found to have the rGly pathway gene set (Figure 2). In contrast to mutually exclusive distribution of the rGly and WL pathways in clade A and B, respectively, both the pathways were estimated to exist in more deeply branching clades C, D, and E compared to clades A and B. Notably, *Desulfoluna butyratoxydans* and *Desulfatirhabdium butyrativorans* in clade C possessed both the gene sets for the WL and rGly pathways. This interspecies distribution of gene sets for the WL and rGly pathways suggested that both the gene sets may have been present in the common ancestor of the phylum *Desulfobacterota*.

### 3.2 Phylogenetic analyses of the enzymes for the WL and rGly pathway

If the common ancestor of the phylum *Desulfobacterota* possessed the gene sets for both the WL and rGly pathways and these genes were vertically inherited, the phylogeny of these genes would be consistent with the phylogeny of species and exhibited the monophyly in the phylum *Desulfobacterota*. Therefore, we conducted phylogenetic analyses focusing on the enzyme genes constituting these pathways to determine whether each enzyme gene was vertically inherited within this phylum or secondarily acquired through horizontal gene transfer (HGT).

**Figure 3:**
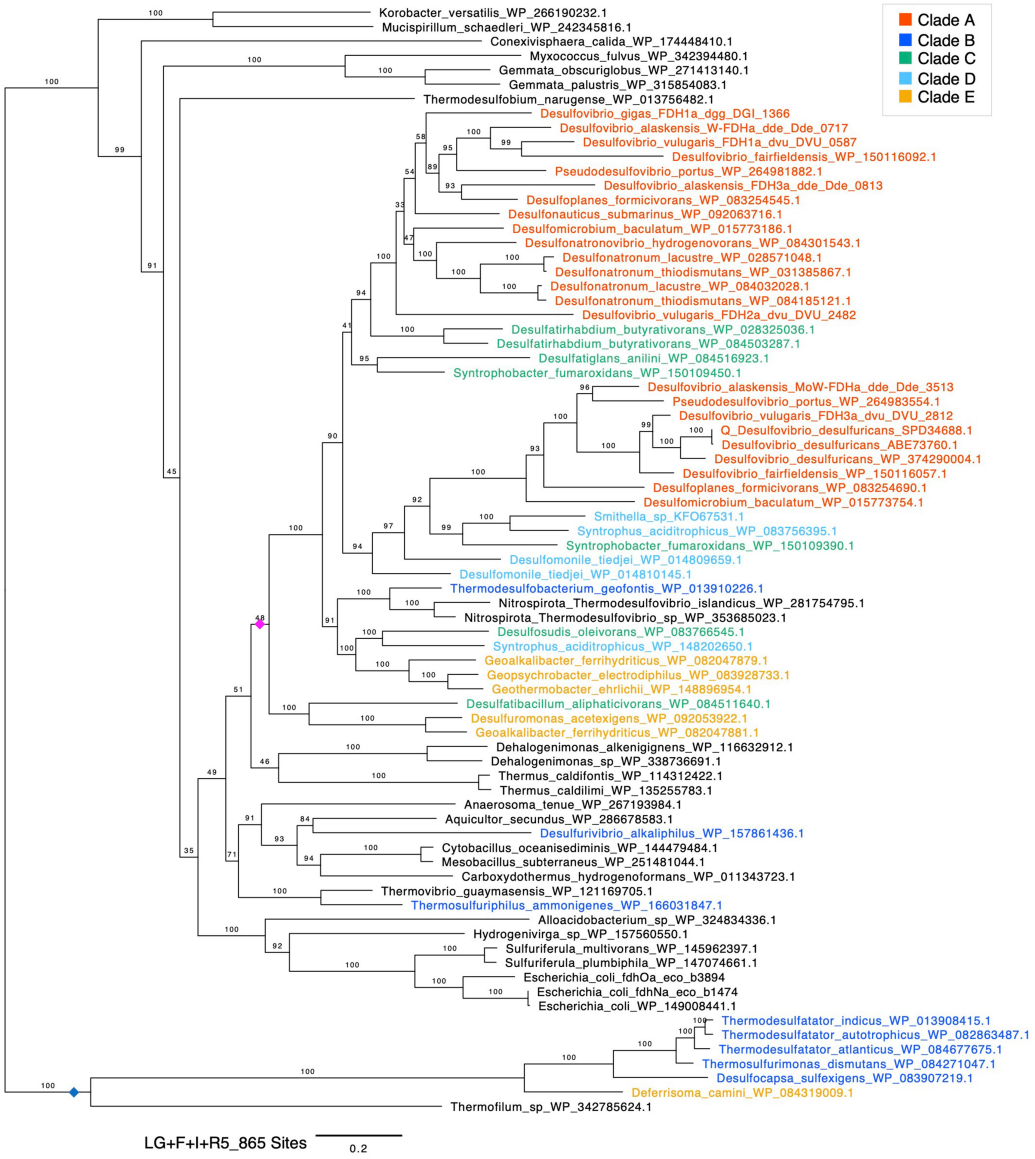
Maximum likelihood phylogenetic tree of FdhG. Bootstrap values were calculated using the ultrafast bootstrap method. The evolutionary model, scale bar and number of sites are shown in the figure. Sequences in the phylum *Desulfobacterota* are highlighted with colors. A pink diamond indicates a clade composed of sequences from *Desulfobacterota* species. A blue diamond indicates clade B and E sequences that were positioned outside the *Desulfobacterota* largest clade.

**Figure 4:**
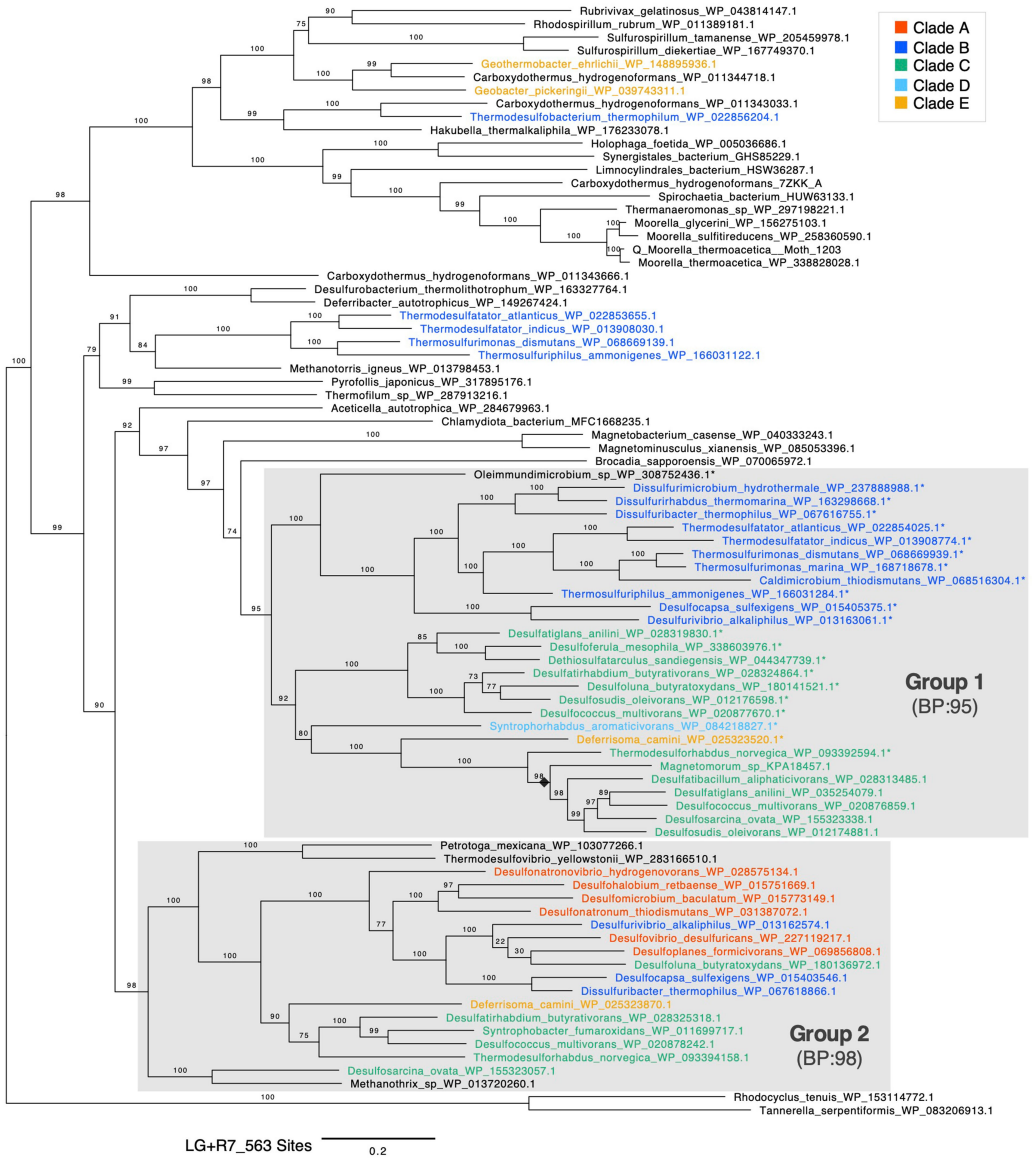
Maximum likelihood phylogenetic tree of AcsA (CODH). Bootstrap values were calculated using the ultrafast bootstrap method. The evolutionary model, scale bar, and number of sites are shown in the figure. Sequences in the phylum *Desulfobacterota* are highlighted with colors. Asterisks (*) indicate sequences encoded in a cluster with other CODH/ACS complex genes. A black diamond indicates a clade within group 1 composed of sequences that do not form a gene cluster.

**Figure 5:**
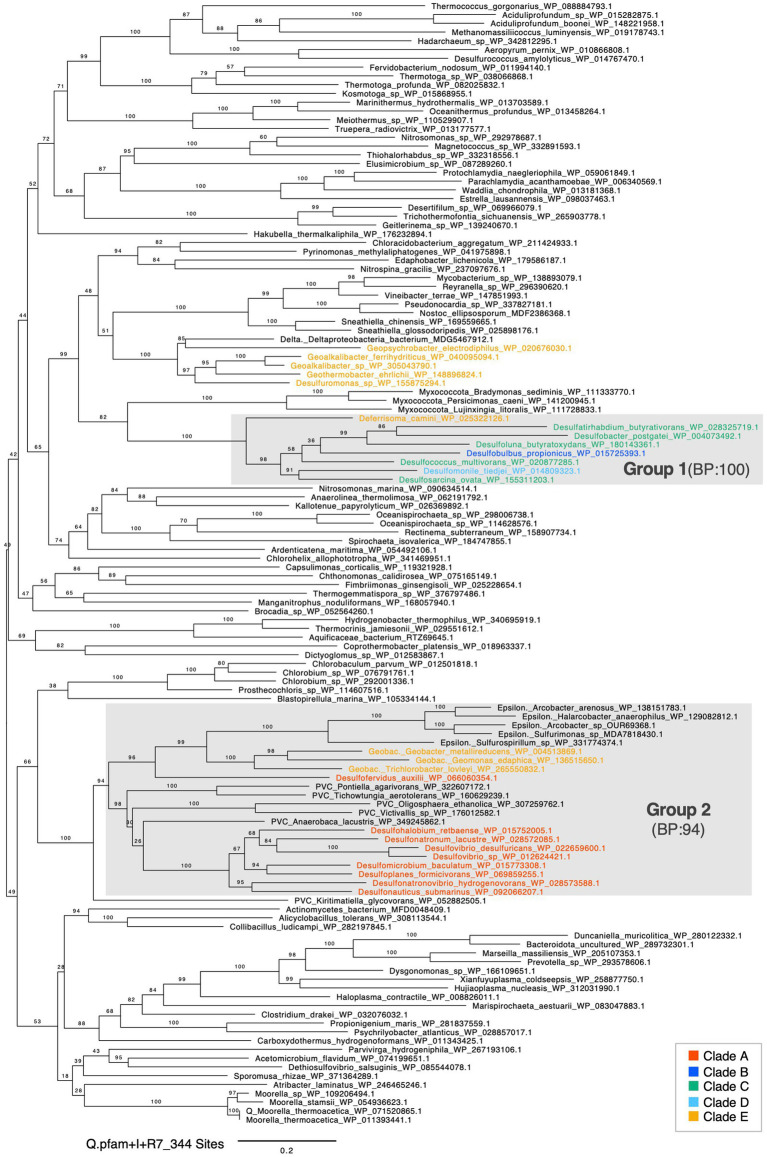
Maximum likelihood phylogenetic tree of GCSP *α*-subunit. Bootstrap values were calculated using the ultrafast bootstrap method. The evolutionary model, scale bar, and number of sites are shown in the figure. Sequences in the phylum *Desulfobacterota* are highlighted with colors.

A total of 28 phylogenetic trees of the enzymes related to the WL pathway, rGly pathway and subsequent amino acid synthesis were inferred (Figures 3–5; Supplementary Figures 3–9). The following section focuses on FDH, CODH/ACS complex, and GCS complex and provides detailed discussions. These three components play particularly important roles: FDH catalyzes the initial step of both the WL and rGly pathways and CODH/ACS and GCS complexes are directly involved in CO_2_ fixation in each pathway.

### 3.3 Phylogenetic analysis of FDHα

In the phylogenetic tree of cytoplasmic Fdh, the sequences from the phylum *Desulfobacterota* did not form a monophyletic group (Supplementary Figure 3A). This result did not support the vertical transmission of cytoplasmic Fdh gene from the common ancestor of the phylum *Desulfobacterota*. In contrast, FdhG from the phylum *Desulfobacterota* formed a monophyletic group (Figure 3, pink diamond). The branching pattern in this monophyletic group was not fully consistent with the phylogenetic relationships of the host species. For instance, sequences from clade A split into two clades. This phylogenetic incongruence can be explained by gene duplications and subsequent gene losses within the phylum *Desulfobacterota*. The presence of sequences derived from the genus *Thermodesulfovibrio*, which belongs to the phylum *Nitrospirota*, is most probably due to HGT from the phylum *Desulfobacterota* to the genus *Thermodesulfovibrio*. Therefore, the phylogenetic tree of FdhG supported the presence of FdhG gene in the common ancestor of the phylum *Desulfobacterota*.

Within the monophyletic group of FdhG in *Desulfobacterota* shown in pink diamond in Figure 3, only one sequence was included from each clade B species, suggesting that FdhG has been lost or undergone substantial genetic divergence in clade B. Two FdhG candidates in clade B organisms (*Desulfurivibrio alkaliphilus* and *Thermosulfuriphilus ammonigenes*) were sporadically distributed outside this monophyletic clade, suggesting the secondary acquisition through HGT. Furthermore, several FdhG sequences from the clade B together with one from the clade E formed a long-branching clade (Figure 3, blue diamond) whose evolutionary origin remains unclear.

### 3.4 Phylogenetic analysis of the CODH/ACS complex

In the phylogenetic tree of AcsA, which encodes CODH, the sequences in *Desulfobacterota* clustered into two distinct clades supported by bootstrap values (BPs) over 95 each (Figure 4, highlighted in gray boxes). These two clades were provisionally designated as group 1 and group 2. Group 1 contained AcsA in *Desulfobacterota* clades B, C, D, and E, including the one in *T. indicus* possessing the functional WL pathway. In contrast, group 2 contained sequences from clades A, B, C, and E. Eight *Desulfobacterota* species encoded two or more AcsA genes within their genomes and each of them were categorized into group 1 and group 2.

The genome synteny analysis of group 1 AcsA revealed that 19 out of 25 *Desulfobacterota*-derived AcsA including the one in *T. indicus* formed a cluster with other CODH/ACS complex subunit genes (Figure 4, highlighted with*), supporting its function as a CODH/ACS complex. The other six AcsA without forming the cluster were grouped into one clade (Figure 4, black diamond). The species with these non-clustered AcsA also retained the previously mentioned AcsA gene that forms the CODH/ACS cluster. Therefore, *Desulfobacterota* species with group 1 AcsA may have functional CODH which works in the WL pathway. In contrast, all the 16 AcsA sequences in group 2 derived from the phylum *Desulfobacterota* did not form the cluster, suggesting that group 2 AcsA is not involved in acetyl-CoA synthesis (Techtmann et al., 2012). This assumption is supported by group 2 AcsA in *D. desulfuricans*, which has only one AcsA and operates the rGly pathway rather than the WL pathway for CO_2_ fixation.

AcsB subunit encodes ACS which catalyzes acetyl-CoA synthesis with CO, a methyl group of CH_3_-THF, and CoA. All the analyzed AcsB in the phylum *Desulfobacterota*, which distributed in all the clades A to E, was clustered into a single clade with a BP support value of 73 (Supplementary Figure 4A). Although the phylogeny of AcsB in the phylum *Desulfobacterota* was not fully consistent with the phylogeny of the host species, sequences in the clades B and C species formed distinct monophyletic groups supported by a BP of 100. Sequences from Chloroflexota (*Dehalogenimonas formicexedens*) and Planctomycetota (*Kuenenia* sp. and *Scalindua japonica*) were most probably the results of HGT from the phylum *Desulfobacterota* to these organisms outside the phylum *Desulfobacterota*. Therefore, the phylogenetic analysis supported the hypothesis that the common ancestor of the phylum *Desulfobacterota* already possessed the AcsB gene. Phylogenetic analyses also indicate that the AcsC and AcsD genes, which encode subunits involved in methyltransferase activity, were likely present in the common ancestor of the phylum *Desulfobacterota*. This is because AcsC and AcsD in the phylum *Desulfobacterota* formed monophyletic clades with BPs over 90, respectively (Supplementary Figures 4C,D). Similar to AcsB, the AcsC and AcsD clades also contained sequences from the Chloroflexota (*Dehalococcoides* sp.) suggesting that gene cluster-level HGT of the CODH/ACS complex may have occurred from the phylum *Desulfobacterota* to the Chloroflexota.

AcsE in the phylum *Desulfobacterota* was dispersed across the phylogenetic tree and did not form a monophyletic group (Supplementary Figure 4D). Therefore, we could not estimate the origin of AcsE in *Desulfobacterota*, which encodes a bacterial-specific subunit involved in THF-corrinoid methyltransferase activity, from the phylogenetic analysis alone.

We also performed a concatenated phylogenetic analysis of the CODH/ACS complex. From the AcsA represented in the single-gene phylogenetic tree, we selected those forming clusters with three or more other Acs subunits and concatenated their sequences. Consequently, concatenated sequences from 20 species were used to construct the phylogenetic tree (Supplementary Figure 11). The topology of the concatenated tree was consistent with that of the single-gene AcsA analyses. Moreover, the bootstrap support (BP) values were higher and the branch lengths shorter in most cases, indicating a more robust phylogenetic reconstruction. These results reinforce both the reliability of the single-gene phylogenetic analyses and the proposed evolutionary scenario.

### 3.5 Phylogenetic analysis of GCS complex

GCSPα in the phylum *Desulfobacterota* was divided into two clades provisionally designated as group 1 and 2, supported by a BP of 100 and 94, respectively (Figure 5). Group 1 GCSPα was comprised of sequences from clades B, C, D, and E and formed a sister group with GCSPα from the phylum *Myxococcota*, the closest relative to the phylum *Desulfobacterota*. In contrast, group 2 GCSPα included sequences from the species belonging to clade A and the species in the order Geobacterales in clade E. This group 2 also contained sequences from outside the phylum *Desulfobacterota*, specifically from Epsilonproteobacteria and the bacterial “PVC group” (Supplementary Figure 2) (Coleman et al., 2021). No species possessed both group 1 and group 2 GCSPα genes.

Similar to GCSPα, the phylogenetic analyses of GCSPβ and GCST clearly divided the *Desulfobacterota* sequences into two groups. Specifically, group 1 consisted of sequences from the *Desulfobacterota* clades B, C, D, and E excluding the order Geobacterales (Supplementary Figures 8A,B), while group 2 consisted of sequences from clade A and the order Geobacterales in clade E, along with sequences from Epsilonproteobacteria and the PVC group.

GCSPα, GCSPβ and GCST categorized into group 1 based on the phylogenetic trees suggested to be vertically inherited from the common ancestor of the phylum *Desulfobacterota* because the sequences from clades B to E formed a monophyletic group although the sequences in the close relatives in the phylum *Myxococcota*. In contrast, group 2 GCSPα, GCSPβ, and GCST were nested within the PVC group lineage, suggesting that the genes encoding these three subunits were most likely horizontally transferred from the PVC group to clade A and the order Geobacterales.

In contrast to the case of GCSPα, GCSPβ, and GCST, GCSL and GCSH in the phylum *Desulfobacterota* were widely scattered across the phylogenetic trees and did not form monophyletic groups. Therefore, we could not estimate the origin of these proteins.

## 4 Discussion

In this study, we investigated the origin and evolutionary history of the WL and rGly pathways in the phylum *Desulfobacterota* by analyzing gene presence/absence and phylogeny of the corresponding enzymes. As a result, we succeeded in inferring the gene sets of the common ancestor of the phylum *Desulfobacterota*, as well as the subsequent processes of gene gain and loss as described below.

The common ancestor of the phylum *Desulfobacterota* is suggested to have possessed the genes for CODH/ACS complex. Four out of five CODH/ACS complex subunits, AcsA, B, C, and D, are inferred to have existed in the common ancestor of the phylum *Desulfobacterota* based on gene presence/absence and phylogenetic analyses. Although our phylogenetic analysis did not reveal the origin of AcsE, this protein is also expected to have existed in the common ancestor of the phylum *Desulfobacterota*. This is because AcsE gene forms a cluster with other CODH/ACS complex genes and is therefore most likely vertically inherited as a cluster. Furthermore, a concatenated phylogenetic analysis of CODH/ACS subunits (AcsA-E) recovered the same topology as the single-gene trees, with higher support, also reinforcing vertical inheritance from the *Desulfobacterota* ancestor (Supplementary Figure 11).

The common ancestor of the phylum *Desulfobacterota* may also have possessed the genes for GCS complex. Both phylogenetic and gene presence/absence analyses supported the vertical inheritance of GCSPα, GCSPβ, and GCST to clade B, C, D and E from the common ancestor of *Desulfobacterota*. In contrast, phylogenetic analyses did not clarify the origin of GCSL and GCSH in the phylum *Desulfobacterota* although they are widely distributed in all the clades (Supplementary Table 6D). This could be explained by rapid sequence evolution caused by low functional constraints or replacement by functionally equivalent proteins because GCSH is a non-catalytic carrier protein and GCSL is an FAD dependent oxidoreductase that re-oxidizes the H-protein (Kikuchi, 1973; Kikuchi et al., 2008). Therefore, we estimated that the common ancestor of *Desulfobacterota* may also have possessed the genes for GCSL and GCSH. To distinguish vertical inheritance from HGT, we compared the gene trees with the species tree and inferred HGT when *Desulfobacterota* sequences were nested within distantly related clades with strong phylogenetic support (e.g., high bootstrap values). In the GCS complex, GCSPα, GCSPβ, and GCST from clade A and from Geobacterales within *Desulfobacterota* are nested within the PVC group (Figure 5; Supplementary Figure 8A). This gene tree topology conflicts with the species tree and constitutes evidence of secondary acquisition via HGT.

The common ancestor of the phylum *Desulfobacterota* is also expected to have FdhG, a catalytic unit of FDH, indicating that it may have all the key enzyme genes for the WL and rGly pathways. Furthermore, presence/absence analysis also detected all the genes other than those for the key enzymes for the WL and rGly pathways in the phylum *Desulfobacterota* (Supplementary Table 6D). Namely, enzymes involved in the methyl branch which are shared between the WL and rGly pathways and an enzyme catalyzing the subsequent reaction in the WL pathway (Supplementary Figure 1) were identified in all the clades. These findings support the possibility that the common ancestor of the phylum *Desulfobacterota* possessed the gene set for the WL and rGly pathways.

**Figure 6:**
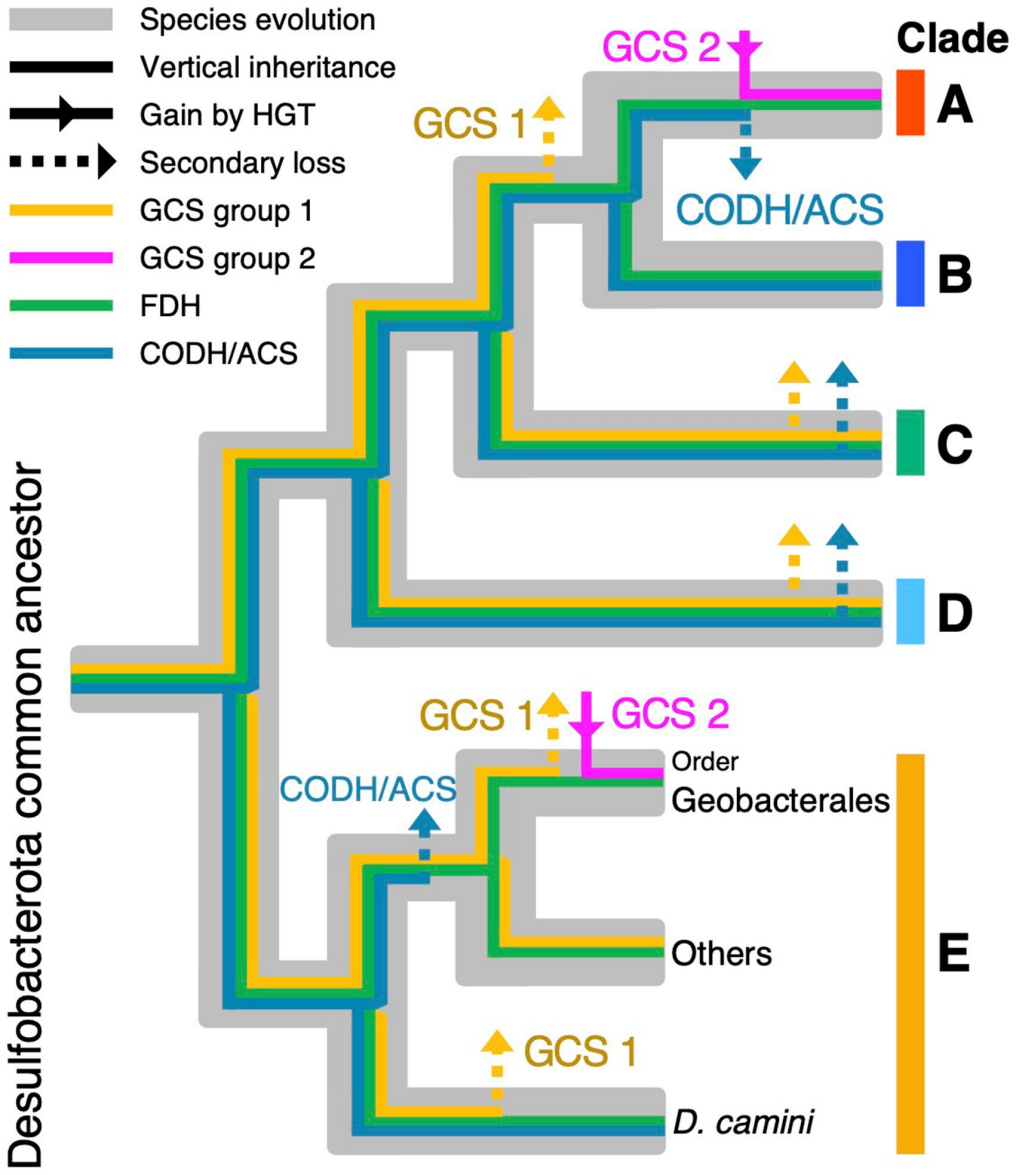
Gain and loss scenarios of gene sets for the WL and rGly pathways in the phylum *Desulfobacterota*. The phylogenetic tree of the phylum *Desulfobacterota* is shown in gray. Presence or absence of FDH, CODH/ACS complex, and GCS complex are shown with color bars explained in the figure.

Based on the phylogeny of enzymes, the following evolutionary scenario is proposed in the phylum *Desulfobacterota* (Figure 6). The gene set for CODH/ACS complex was lost in the common ancestor of clade A and in clade E after the ancestor of *D. camini* was separated from others (Figure 6, Blue). GCS complex genes were lost in the common ancestor of clades A-B and in the order Geobacterales within clade E (Figure 6, Yellow). The CODH/ACS complex and/or GCS complex have also been lost in some species in clades B to E multiple times. Moreover, clade A and the order Geobacterales regained group 2 GCSPα, Pβ, and T from PVC group via HGT (Figure 6, Pink). These findings indicate that individual lineages have formed unique gene repertoires through gene losses and acquisitions via HGT.

Although the common ancestor of *Desulfobacterota* is expected to have possessed all the enzyme genes for the WL and rGly pathways, it remains unsolved whether these genes have worked for CO_2_ fixation. To estimate their function for autotrophic growth, we analyzed downstream of the carbon fixation pathways. If the common ancestor of the phylum *Desulfobacterota* grew autotrophically via the WL pathway, it would have pyruvate ferredoxin oxidoreductase, phosphoserine phosphatase, and serine hydroxymethyltransferase (Figure 1), because these enzymes are essential to synthesize serine and glycine from acetyl-CoA through pyruvate. As a result, the three enzymatic genes were detected in all clades of the phylum *Desulfobacterota* (Supplementary Table 6D). Although phylogenetic analyses of these three enzymes did not support their vertical inheritance from the common ancestor of the phylum *Desulfobacterota* (Supplementary Figures 5–7) plausibly due to frequent HGT and gene duplication events, these were likely present in the common ancestor of *Desulfobacterota*. Therefore, the common ancestor of *Desulfobacterota* may have been capable of autotrophic growth via the WL pathway.

We also analyzed the five subunits of the glycine reductase complex (Figure 1), which catalyzes the conversion of glycine to acetyl-CoA via acetyl phosphate and therefore reported to be essential for autotrophic growth via the rGly pathway (Sánchez-Andrea et al., 2020; Song et al., 2020). Neither gene presence/absence nor phylogenetic analysis supported the presence of the glycine reductase complex in the common ancestor. Still, there remains a possibility that glycine produced via the rGly pathway had been converted to serine by SHMT and then to pyruvate. The common ancestor of the phylum *Desulfobacterota* might have grown autotrophically by operating both the WL and rGly pathways like *C. drakei* (Song et al., 2020). Therefore, *Desulfobacterota* might have been capable of autotrophic growth via the rGly pathway.

To date, no species within the phylum *Desulfobacterota* has been reported to operate both the WL and rGly pathways. Our presence/absence analysis demonstrates that two species in clade C (*Desulfoluna butyratoxydans* and *Desulfatirhabdium butyrativorans*) possess the key gene sets for both the WL and rGly pathways (Figure 2), however, both species are known as heterotrophs (Suzuki et al., 2008; Balk et al., 2008). Further studies are needed to address the possibility that they grow autotrophically under unknown conditions and/or these metabolic pathways function as auxiliary pathways for organic compound acquisition or as sink for reducing power (Schneeberger, 1999; Sánchez-Andrea et al., 2020).

LBCA is estimated to have a gene set for the rGly pathway except for FDH (Coleman et al., 2021). However, their study used only cytoplasmic Fdh (K05299, K15022) as the query for FDH based on the information of KEGG pathway map, in which only cytoplasmic Fdh is registered (Aug 2025). Nonetheless, FDH has multiple isomers other than cytoplasmic Fdh, including FdhG that converts CO_2_ to formate in the rGly pathway of *D. desulfuricans* (Wells et al., 2023). Therefore, it remains possible that the LBCA did possess FDH that is not classified as cytoplasmic Fdh, like the case of the common ancestor of the phylum *Desulfobacterota* which may have possessed FdhG. In future work, comparative analyses of predicted tertiary structures of key enzymes and complexes, including cytoplasmic FDH and FdhG, will be important. Such structure-level comparisons may help clarify how structural diversification of these enzymes is related to the differential use of the WL and rGly pathways and to environmental adaptation within this group.

In this study, we focused on a specific phylum and estimated the metabolisms of its last common ancestor. This phylum-specific studies ensures appropriate taxon sampling and enables more accurate inference of ancestral traits than the studies focused on deeper lineage, such as LBCA (Superson et al., 2019; Powell and Battistuzzi, 2022). Accumulation of knowledge based on such clade-specific studies across various phyla will lead to a more precise inference of metabolism in ancestral organisms such as LBCA and LUCA.

## Data Availability

The original contributions presented in the study are included in the article/Supplementary material, further inquiries can be directed to the corresponding author.
