# Supplementary material for: Molecular evolution of the Wood-Ljungdahl pathway and the reductive glycine pathway in Desulfobacterota
Source: Front Microbiol. 2026 Jan 9;16:1708584. doi: 10.3389/fmicb.2025.1708584 (PMC12827605; doi:10.3389/fmicb.2025.1708584)
Supplement: Supplementary file 7 [file Data_Sheet_1.pdf]

Supplementary Material

Molecular evolution of the Wood-Ljungdahl pathway and the reductive glycine pathway in Desulfobacterota

Tomoyuki Wakashima, Keitaro Kume, Yoko Chiba*

*** Correspondence:** Yoko Chiba: yoko.chiba.ey@a.riken.jp

1 Supplementary Tables

Supplementary Table 1. Subunit composition of CODH/ACS complex.
Presented as a separate MS Excel file.

Supplementary Table 2. Culture conditions.
Presented as a separate MS Excel file.

Supplementary Table 3. Queries used for presence/absence analyses.
Presented as a separate MS Excel file.

Supplementary Table 4. Queries and summary for phylogenetic analyses.
Presented as a separate MS Excel file.

Supplementary Table 5. NCBI Taxonomy IDs for phylogenetic analyses.
Presented as a separate MS Excel file.

Supplementary Table 6. Results of gene presence/absence analyses.
Presented as a separate MS Excel file.

2 Supplementary Data

All FASTA files of alignment data used to infer the phylogenetic trees.

3 Supplementary Figures

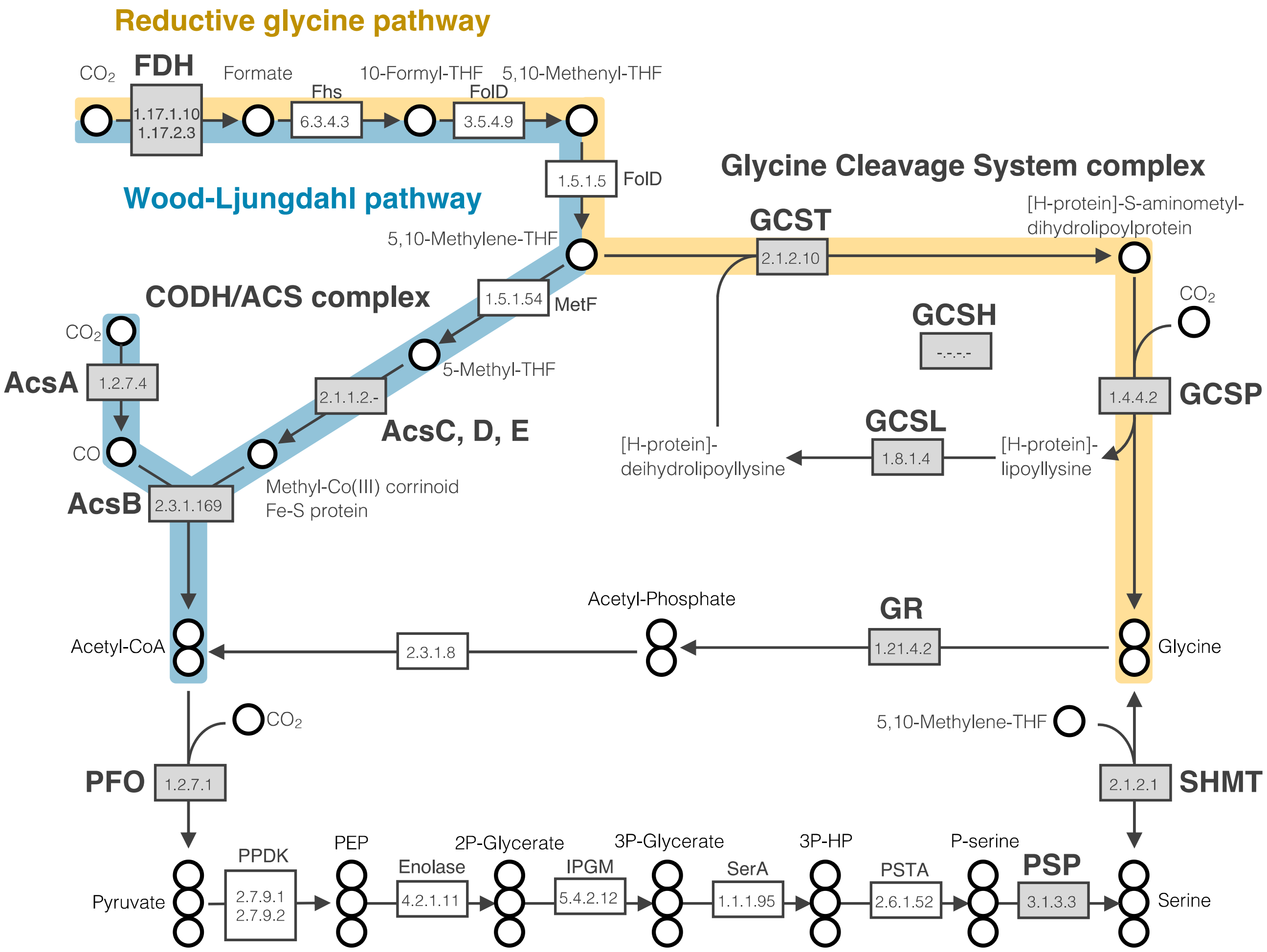

Supplementary Figure 1. The WL and rGly pathways and related metabolism.
Each arrow represents an enzymatic reaction and box indicates the EC number. Gray boxes highlighted enzymes subjected to phylogenetic analysis. Enzyme names are shown in bold. Circles represent carbon. THF: tetrahydrofolate; FDH: formate dehydrogenase; CODH/ACS: carbon monoxide dehydrogenase/acetyl-CoA synthase; GCS: glycine cleavage system; GCST: glycine cleavage system T-protein; GCSP: glycine cleavage system P-protein; GCSL: glycine cleavage system L-protein; GCSH: glycine cleavage system H-protein; PFO: pyruvate-ferredoxin oxidoreductase; SHMT: serine hydroxymethyltransferase; PSP: phosphoserine phosphatase; GR: glycine reductase; PEP: phosphoenolpyruvate; 2P-Glycerate: 2-phosphoglycerate; 3P-Glycerate: 3-phosphoglycerate; 3P-HP: 3-phosphohydroxypyruvate; P-serine: phosphoserine.

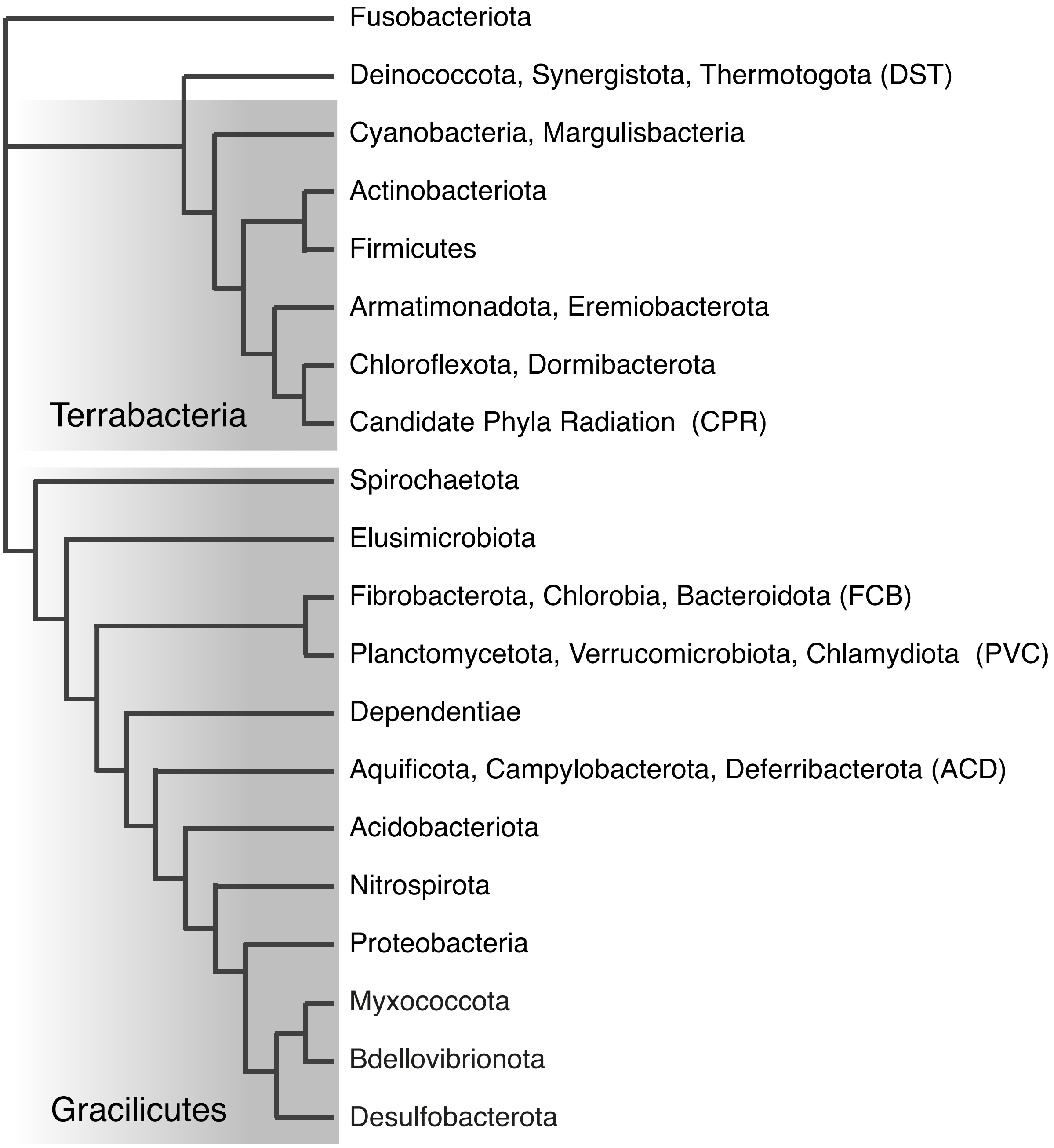

Supplementary Figure 2. Unrooted phylogenetic tree of the bacterial domain.
An unrooted phylogenetic tree of the bacterial domain based on Coleman et al. and Waite et al.

【Fig. S3A. Maximum likelihood phylogenetic tree of cytoplasmic Fdh α-subunit.】

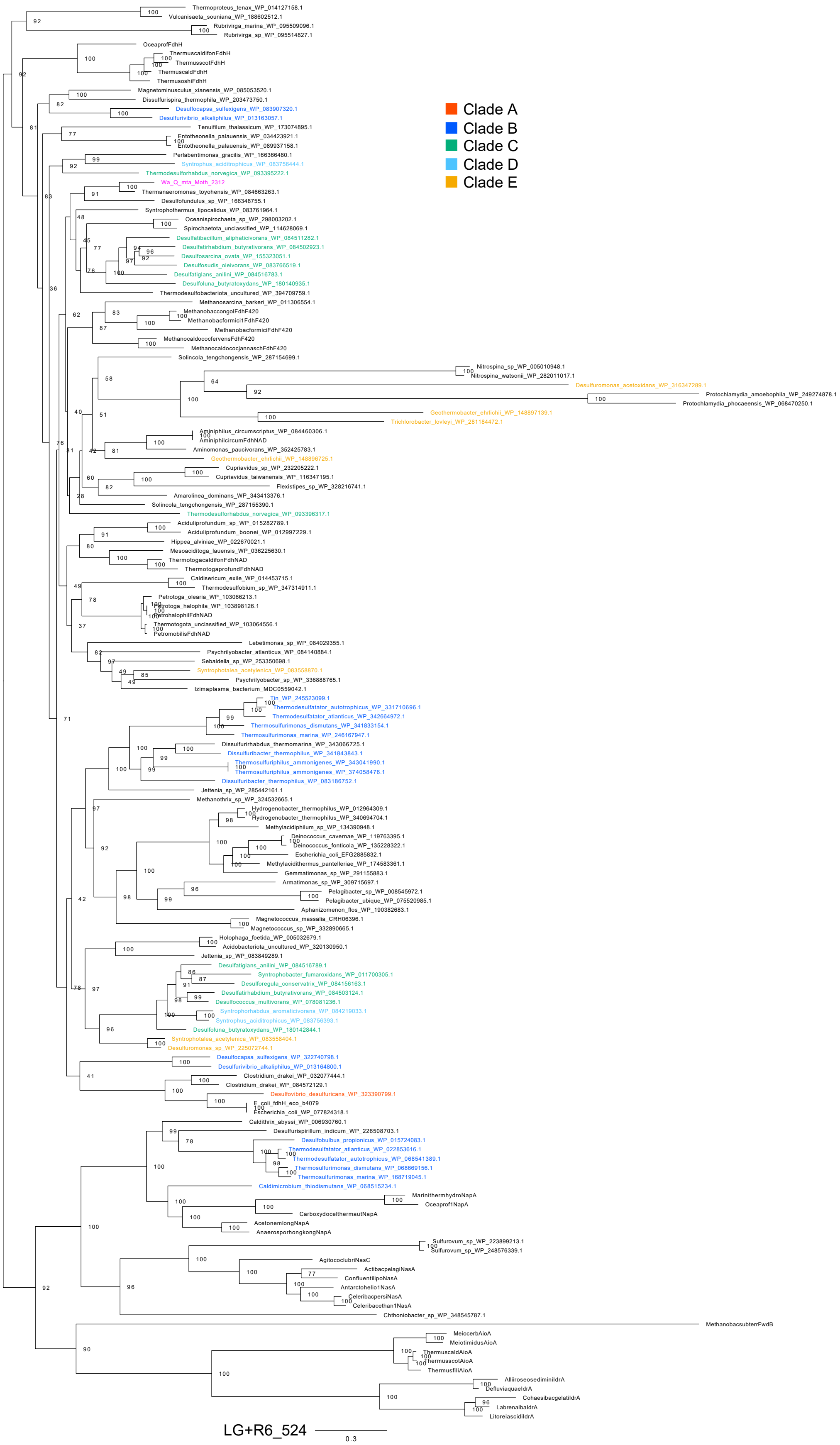

【Fig. S3B. Maximum likelihood phylogenetic tree of cytoplasmic Fdh β-subunit.】

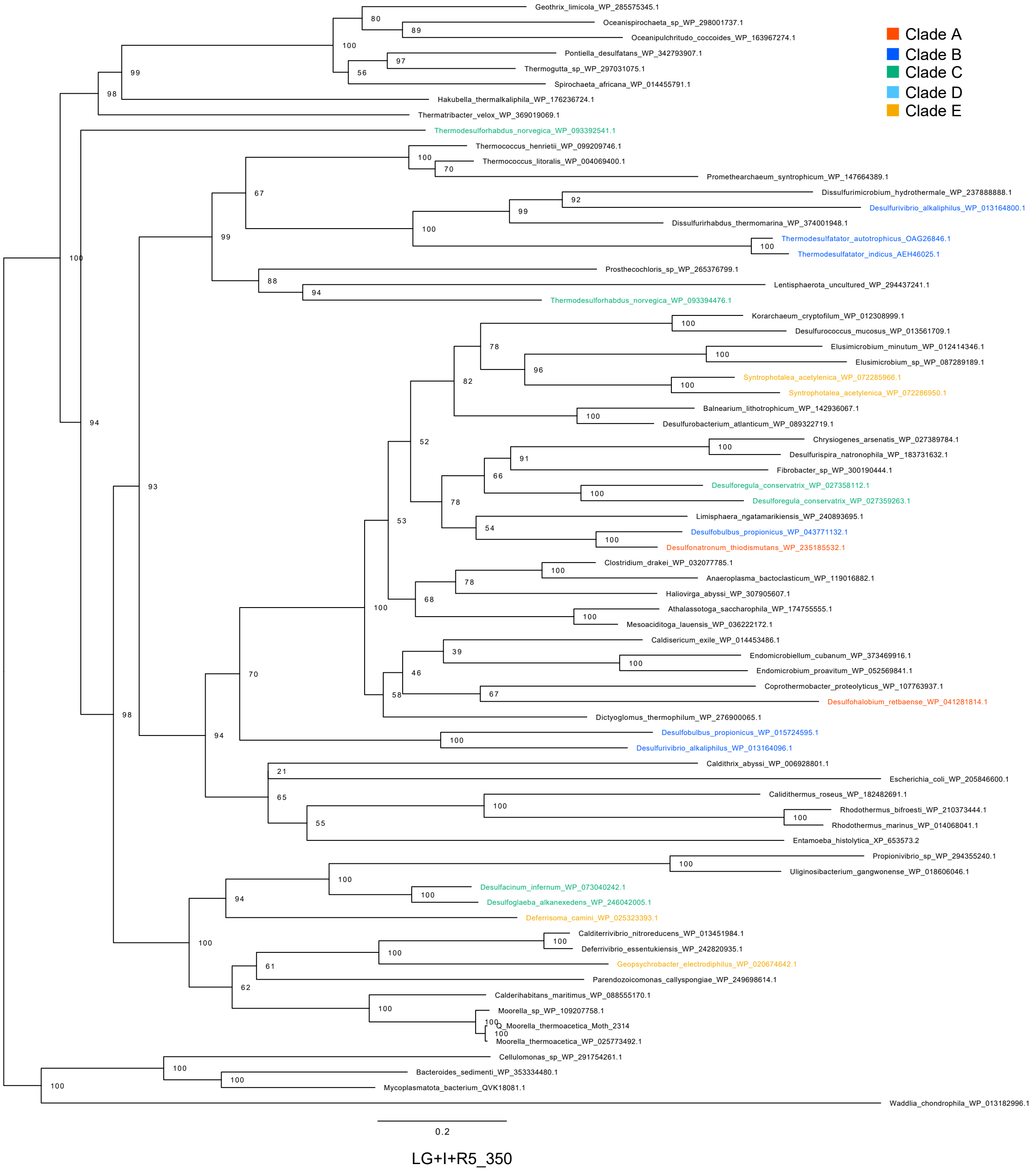

【Fig. S3C. Maximum likelihood phylogenetic tree of FdhG β-subunit.】

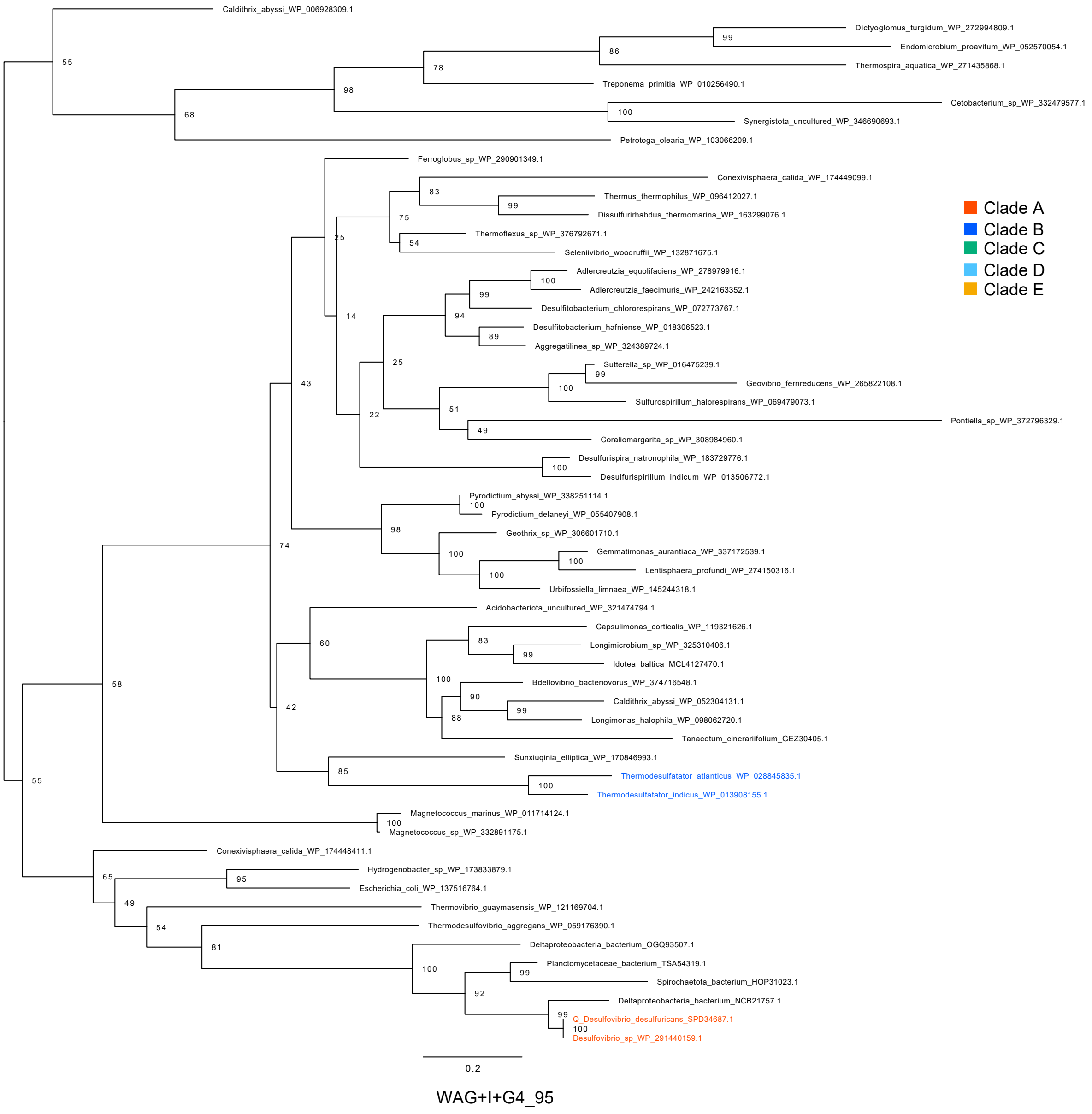

【Fig. S3D. Maximum likelihood phylogenetic tree of FdhG γ-subunit.】

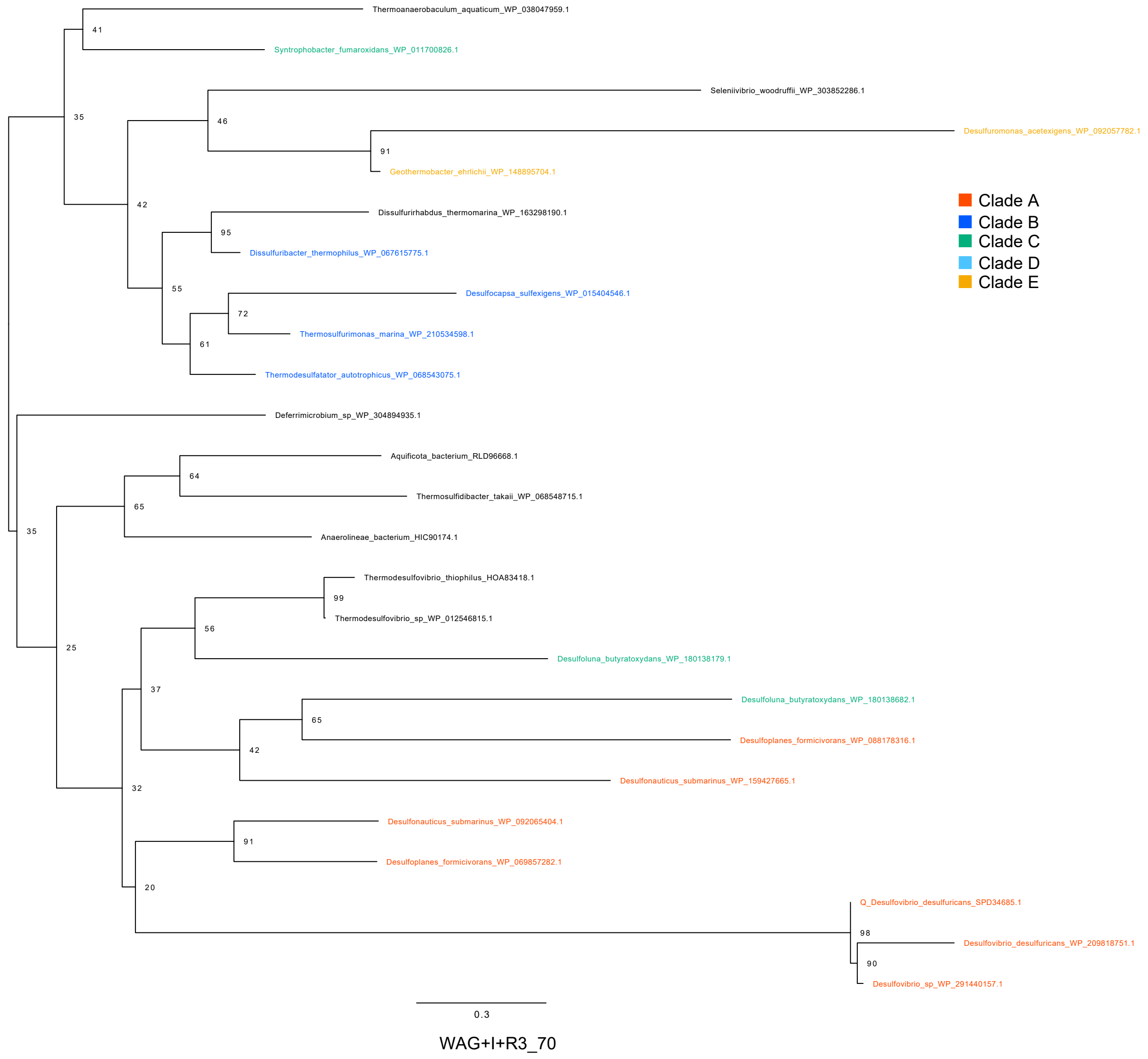

Supplementary Figure 3. Maximum likelihood phylogenetic tree of FDH.
(A) Cytoplasmic Fdh α-subunit; (B) Cytoplasmic Fdh β-subunit; (C) FdhG β-subunit; (D) FdhG γ-subunit. Bootstrap values were calculated using the ultrafast bootstrap method. The evolutionary model, scale bar and number of sites are shown in the figure. Sequences in the phylum Desulfobacterota are highlighted with colors.

【Fig. S4A. Maximum likelihood phylogenetic tree of AcsB.】

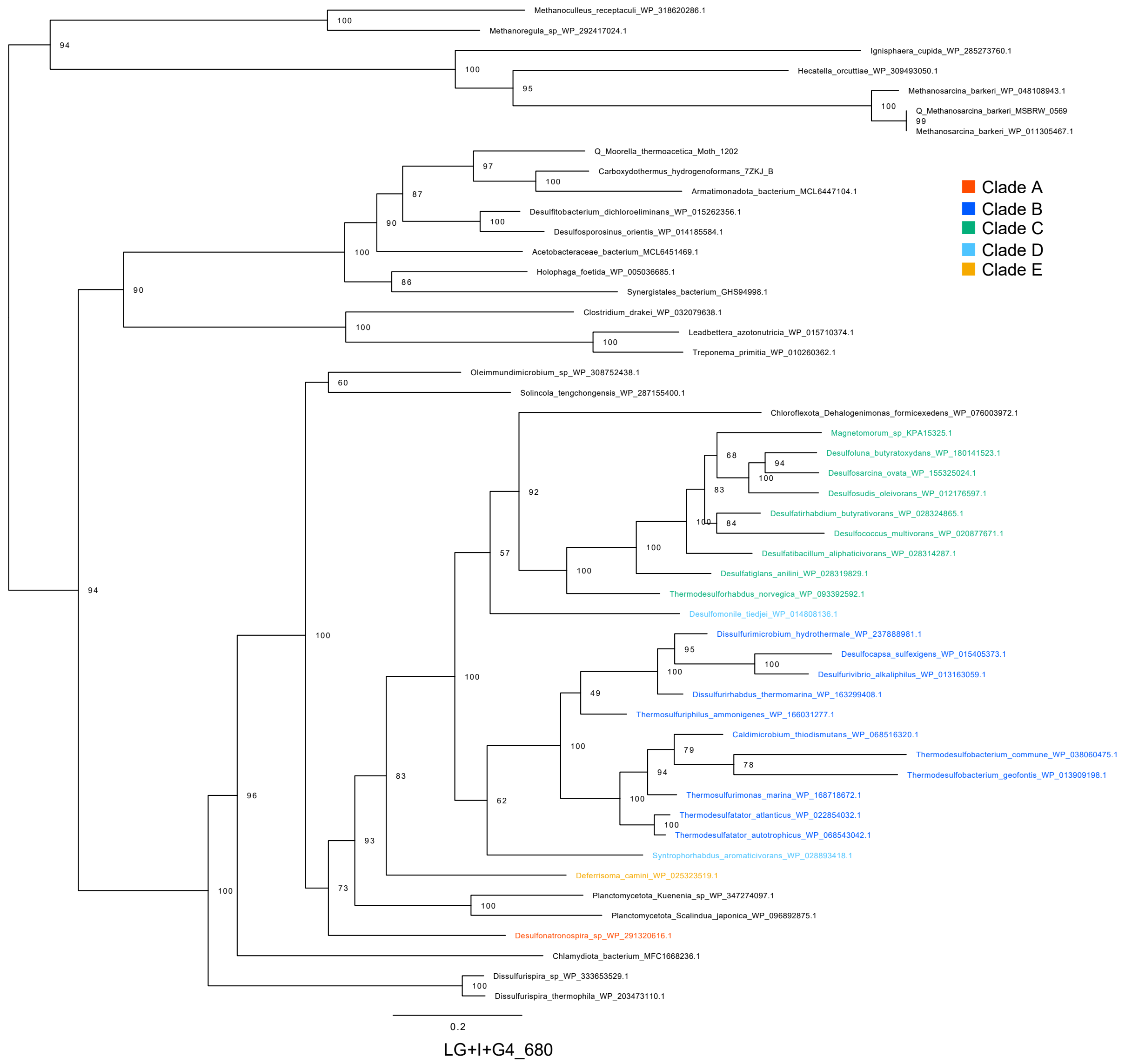

【Fig. S4B. Maximum likelihood phylogenetic tree of AcsC.】

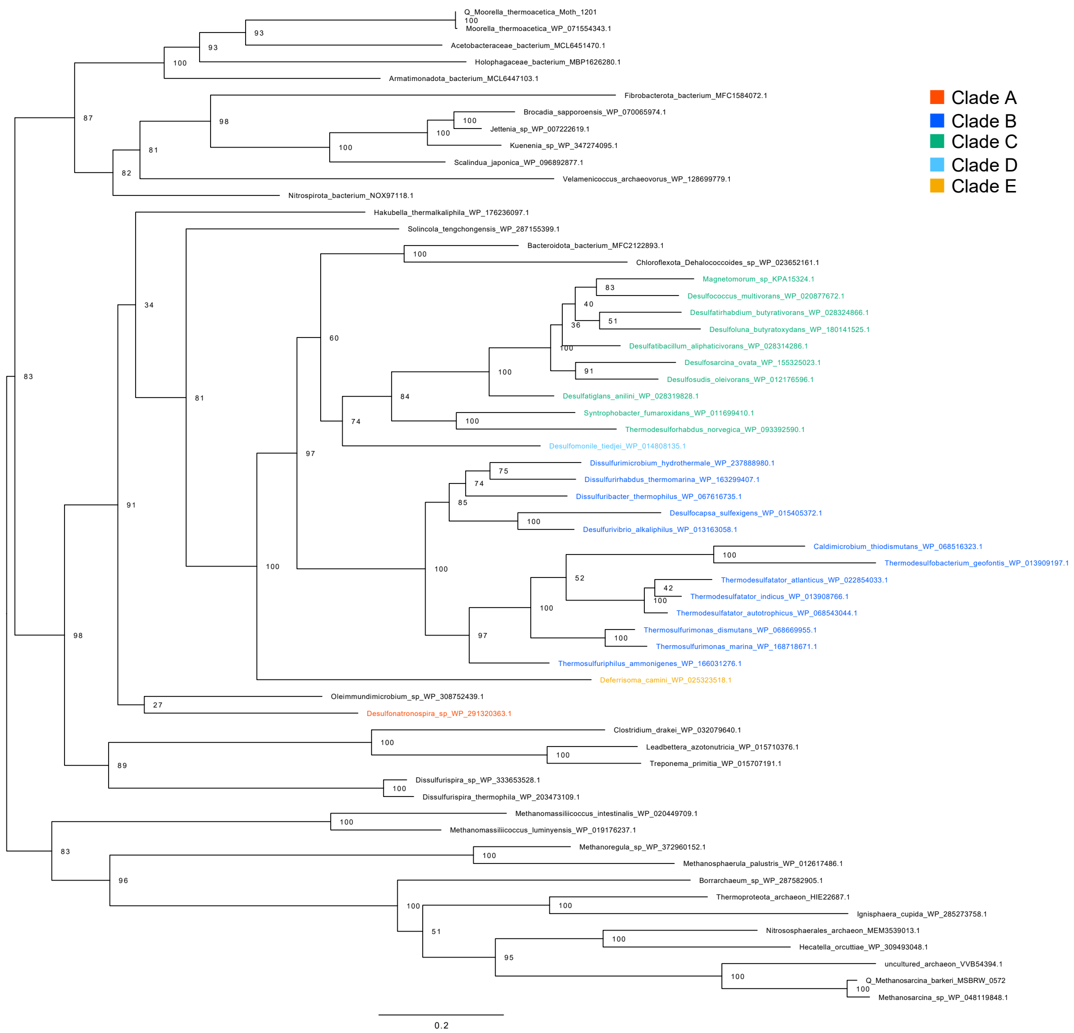

LG+F+I+G4_402

【Fig. S4C. Maximum likelihood phylogenetic tree of AcsD.】

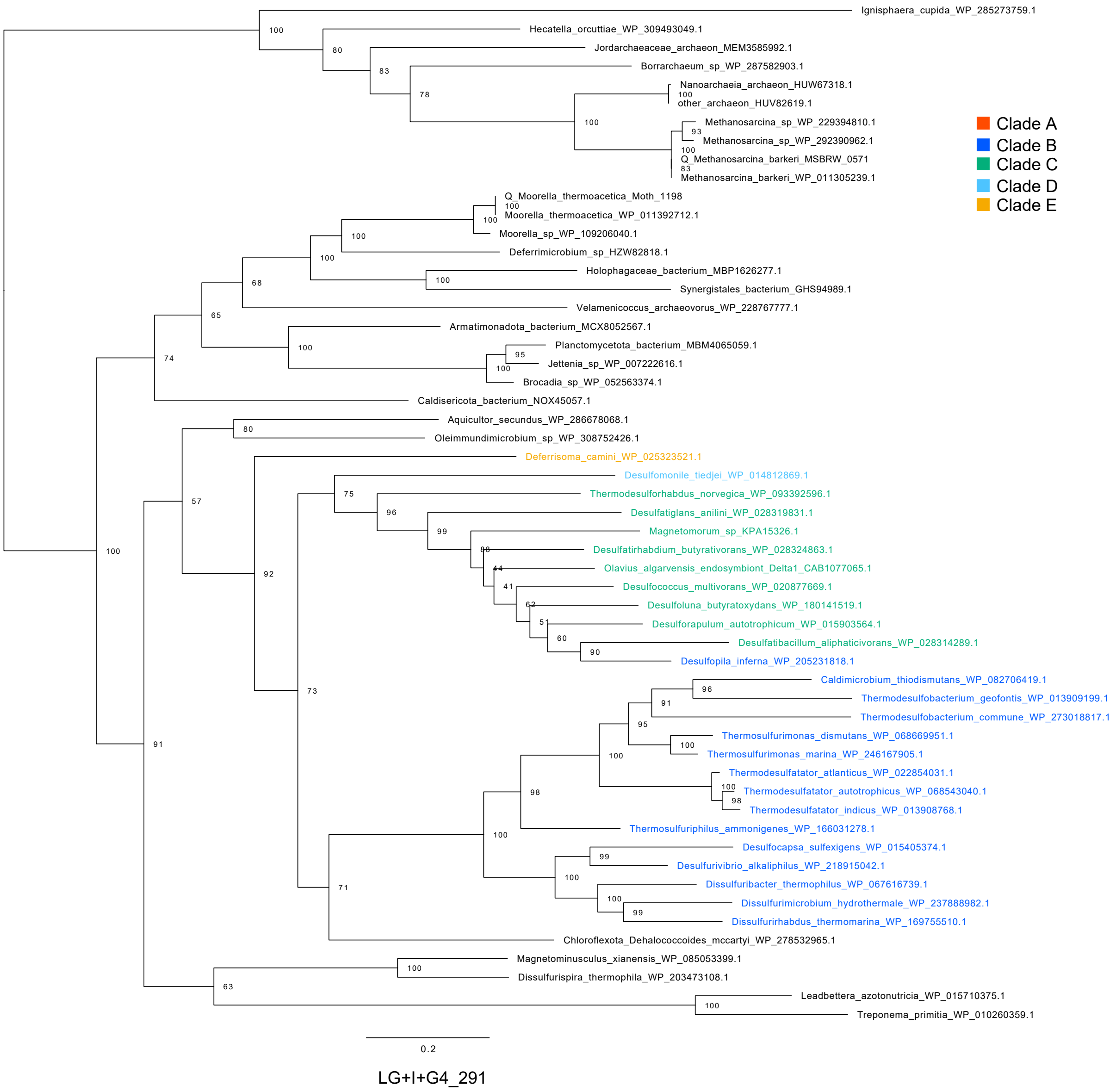

)

【Fig. S4D. Maximum likelihood phylogenetic tree of AcsE.】

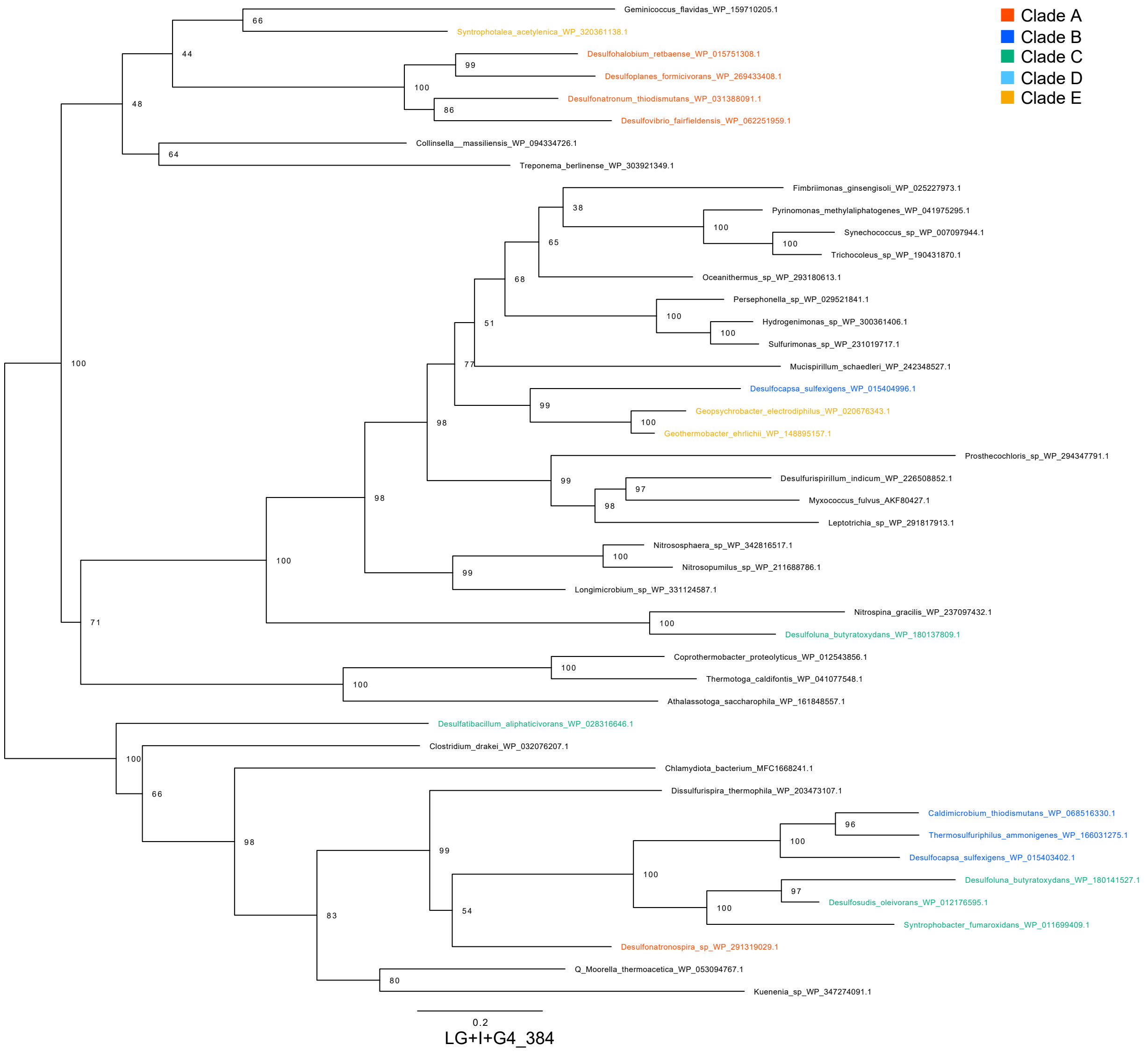

Supplementary Figure 4. Maximum likelihood phylogenetic tree of CODH/ACS complex. (A) AcsB; (B) AcsC; (C) AcsD; (D) AcsE. Bootstrap values were calculated using the ultrafast bootstrap method. The evolutionary model, scale bar and number of sites are shown in the figure. Sequences in the phylum Desulfobacterota are highlighted with colors.

【Fig. S5A. Maximum likelihood phylogenetic tree of PFO (fused).】

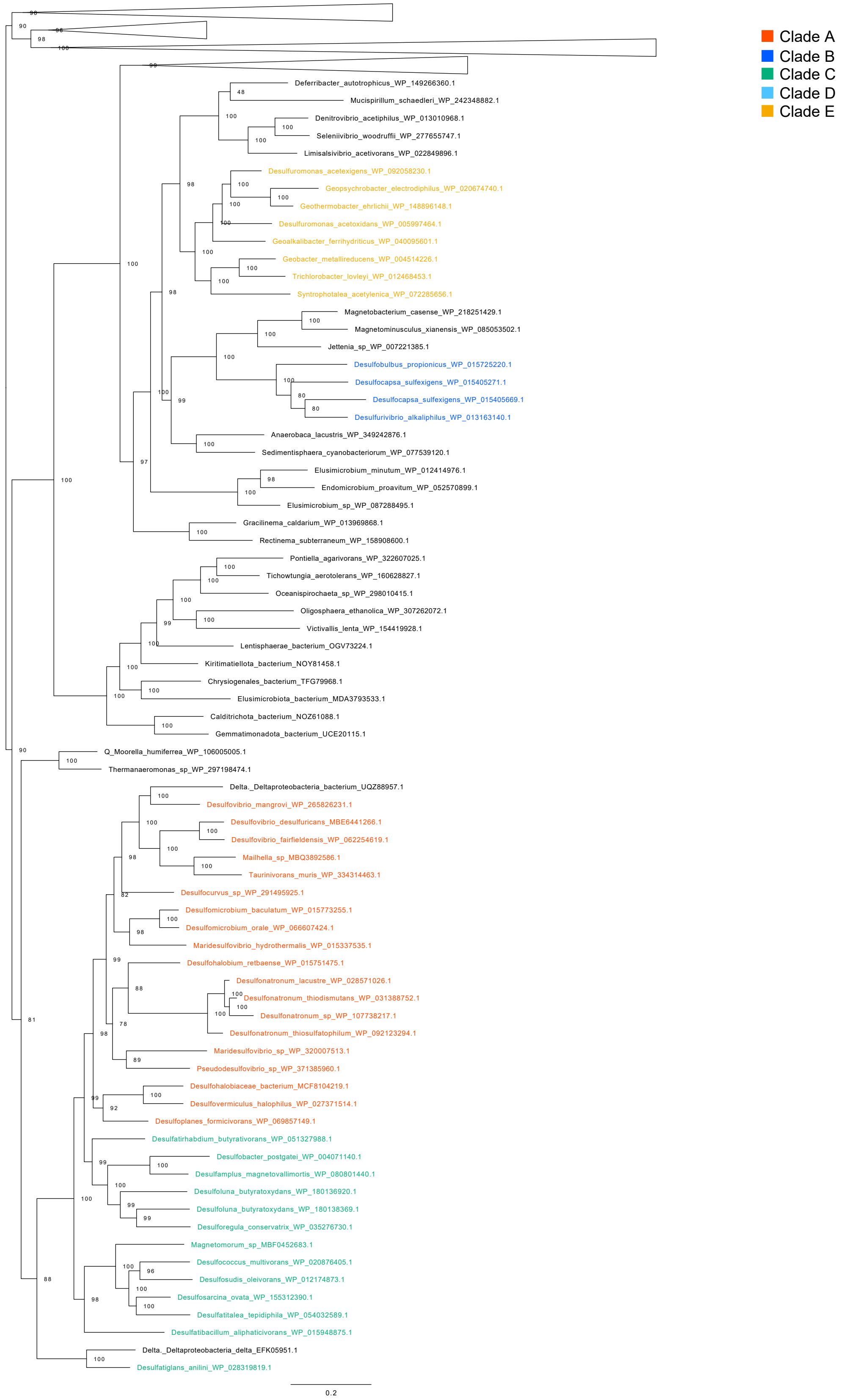

LG+F+I+R10_933

【Fig. S5B. Maximum likelihood phylogenetic tree of PFO α-subunit.】

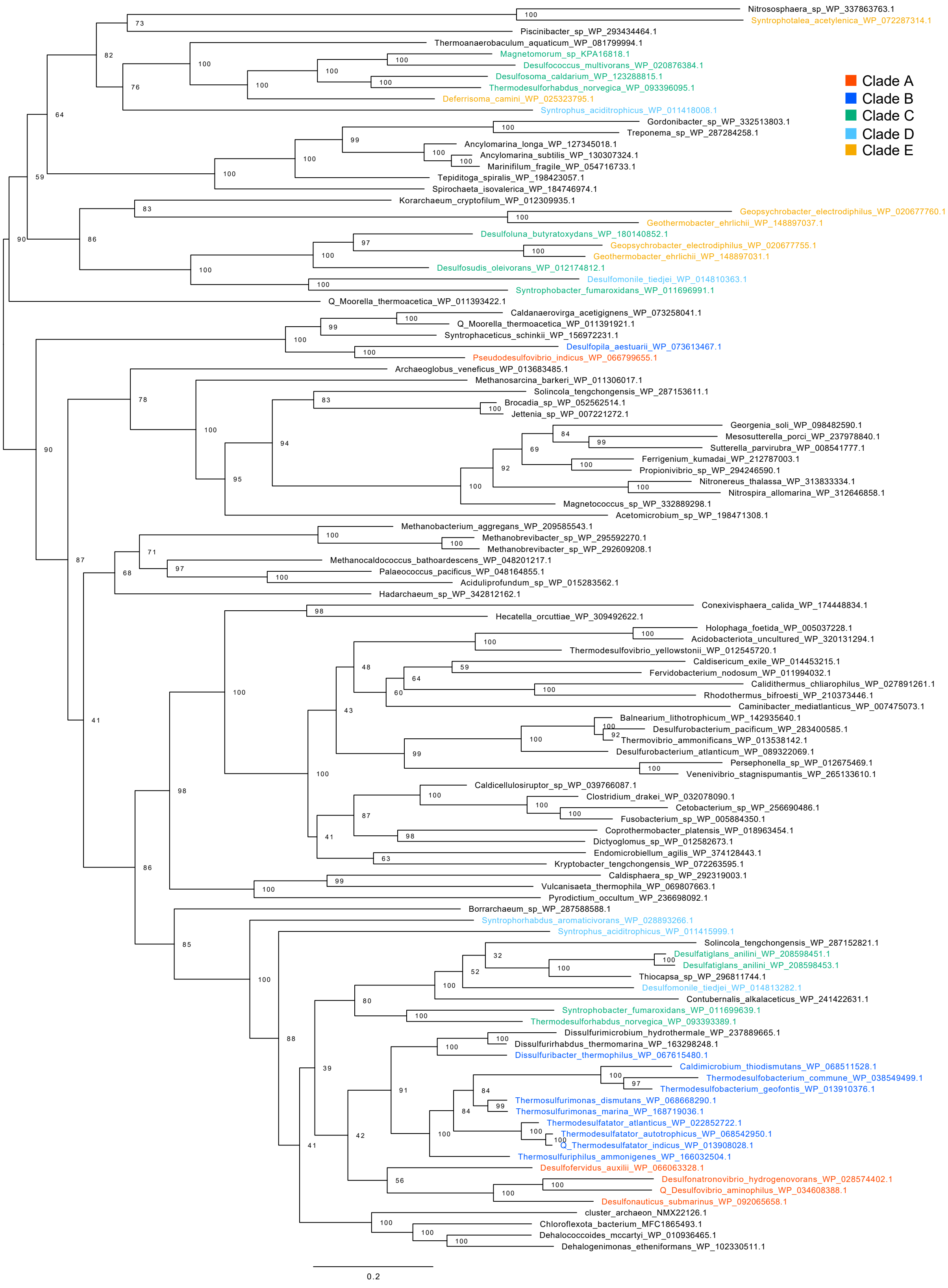

LG+F+I+R5_321

【Fig. S5C. Maximum likelihood phylogenetic tree of PFO β-subunit.】

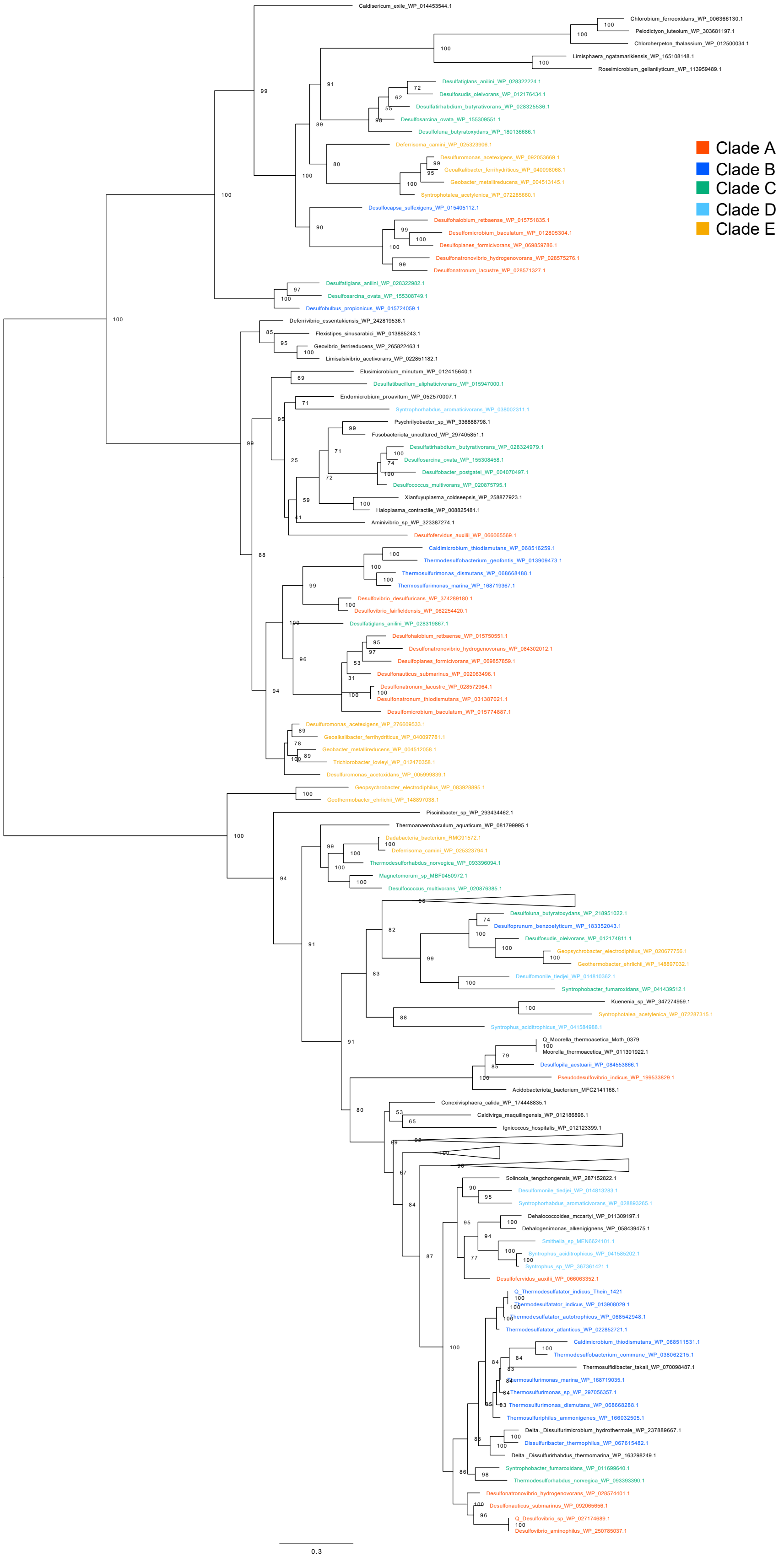

LG+I+R7_182

【Fig. S5D. Maximum likelihood phylogenetic tree of PFO γ-subunit.】

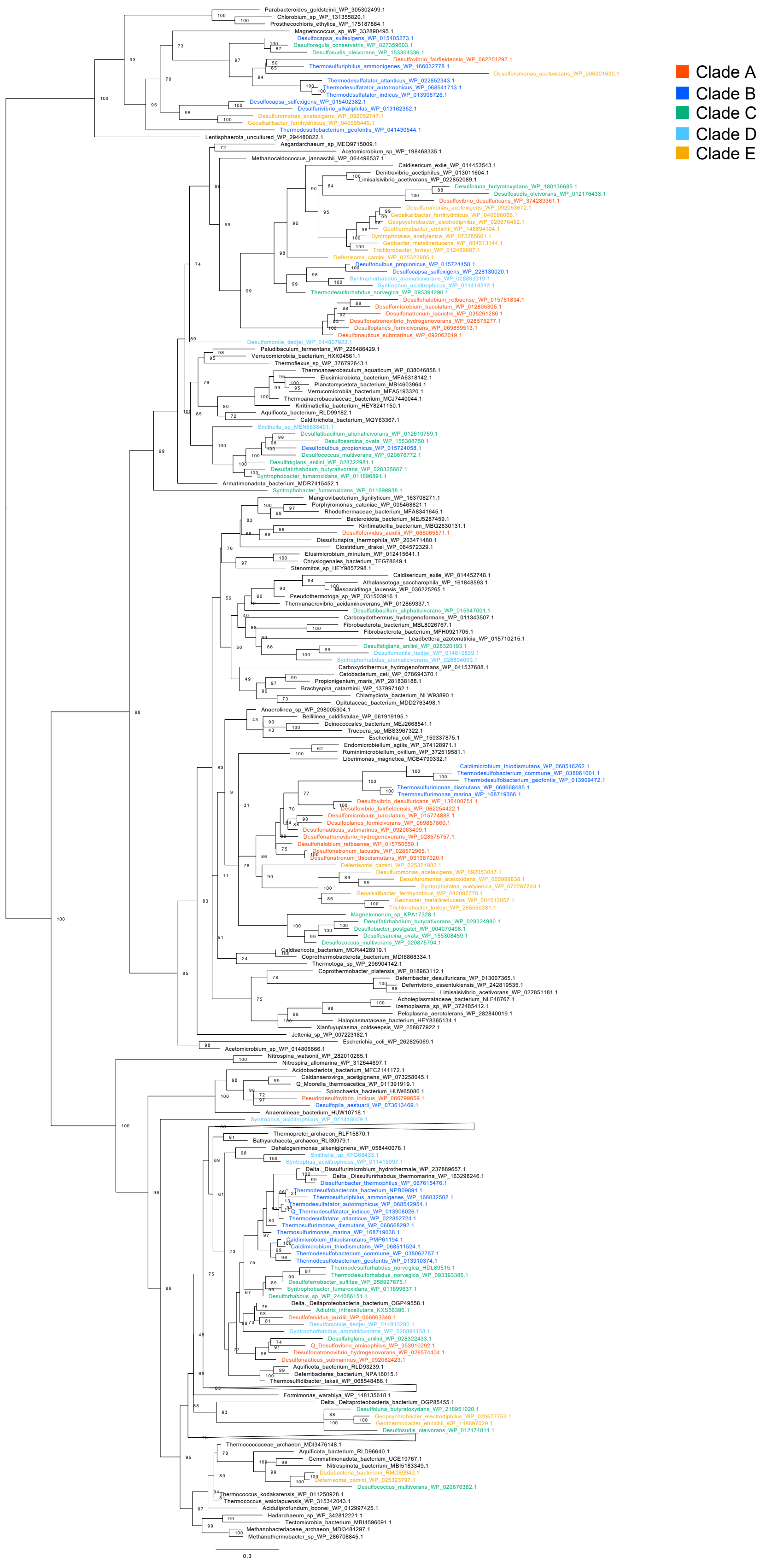

LG+I+R5_93

Supplementary Figure 5. Maximum likelihood phylogenetic tree of PFO.
(A) PFO (fused); (B) PFO α-subunit; (C) PFO β-subunit; (D) PFO γ-subunit.
Bootstrap values were calculated using the ultrafast bootstrap method. The evolutionary model, scale bar and number of sites are shown in the figure. Sequences in the phylum Desulfobacterota are highlighted with colors.

【Fig. S6A. Maximum likelihood phylogenetic tree of dPSP1.】

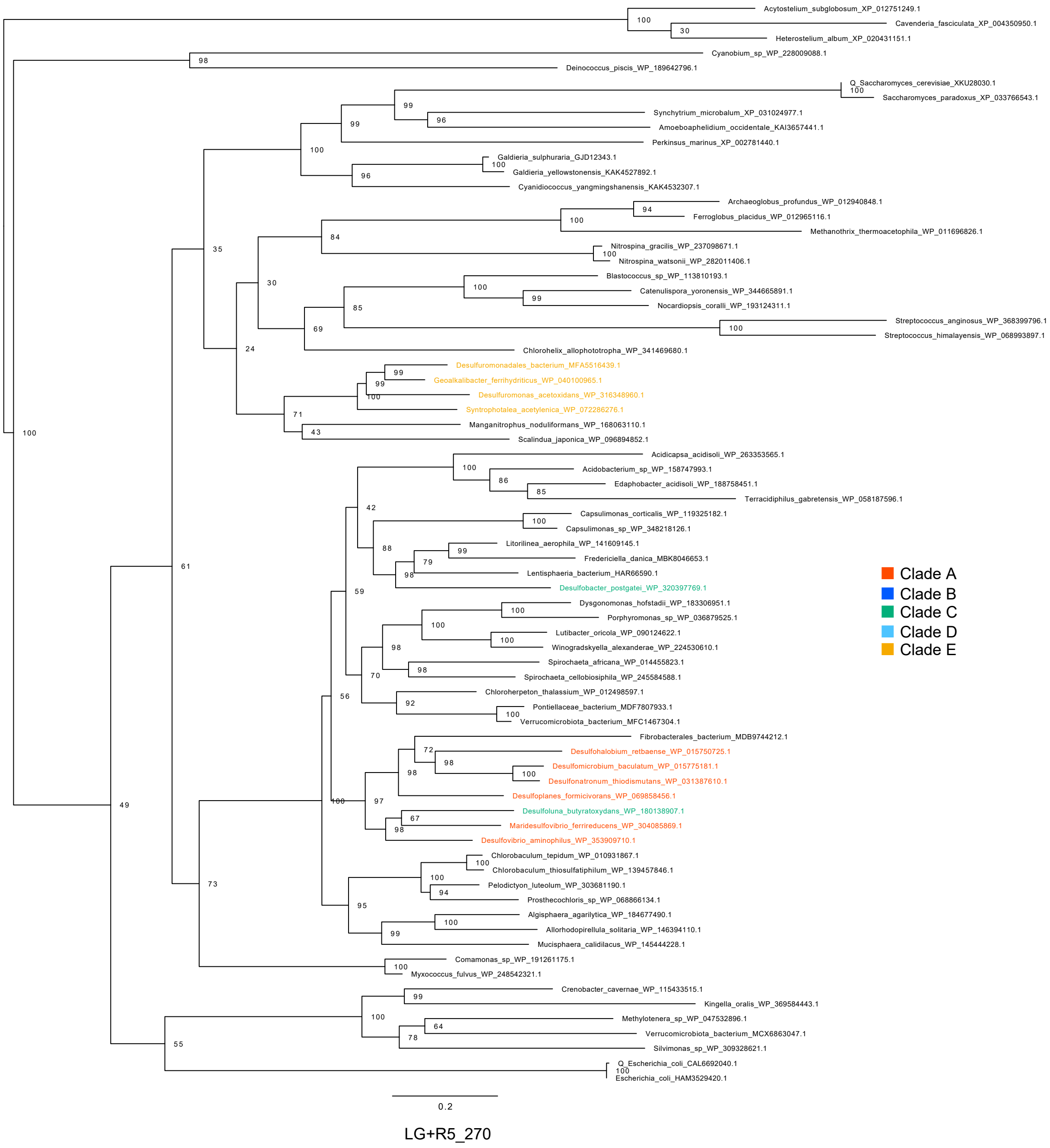

【Fig. S6B. Maximum likelihood phylogenetic tree of dPSP2.】

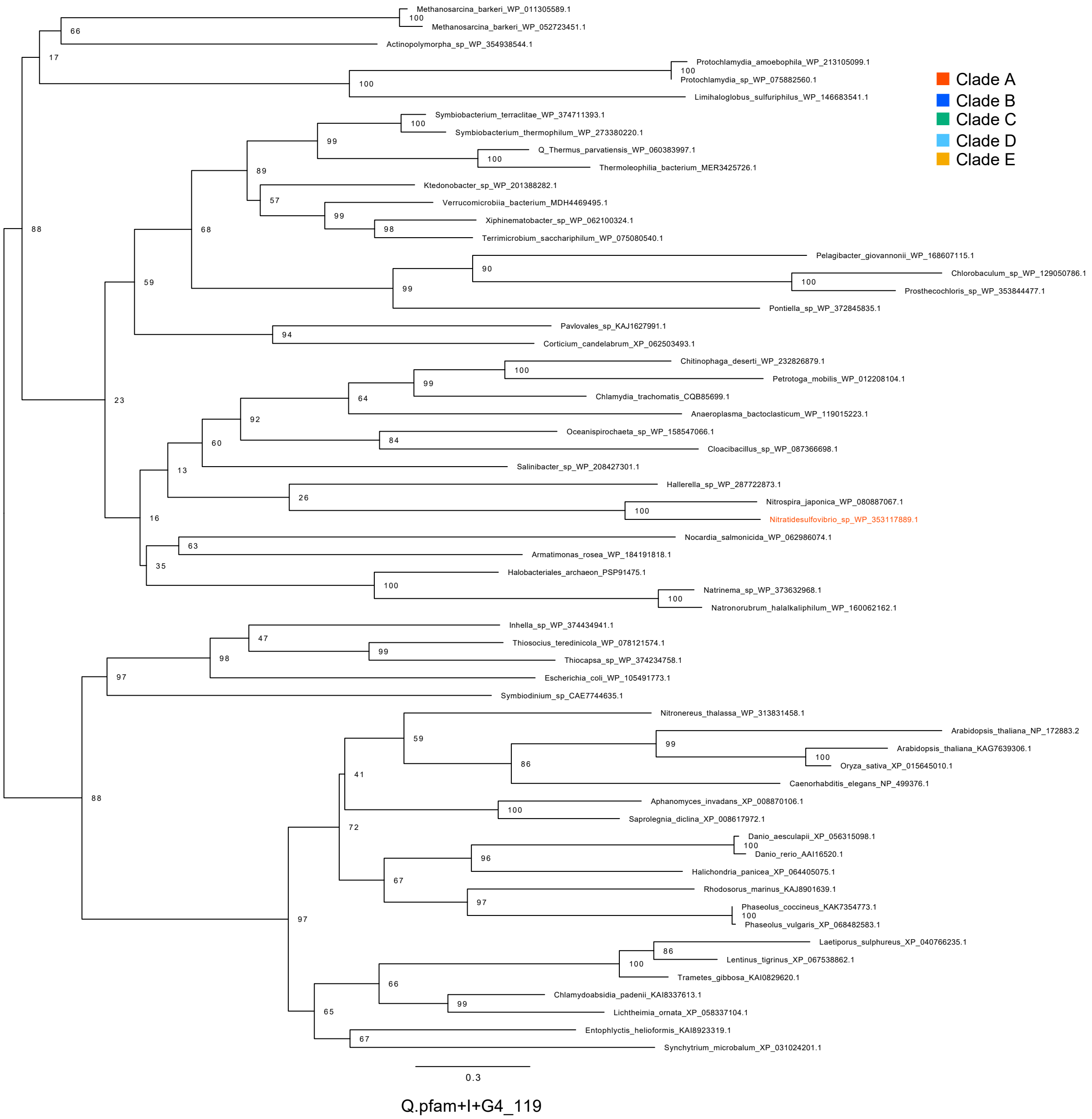

【Fig. S6C. Maximum likelihood phylogenetic tree of iPSP1.】

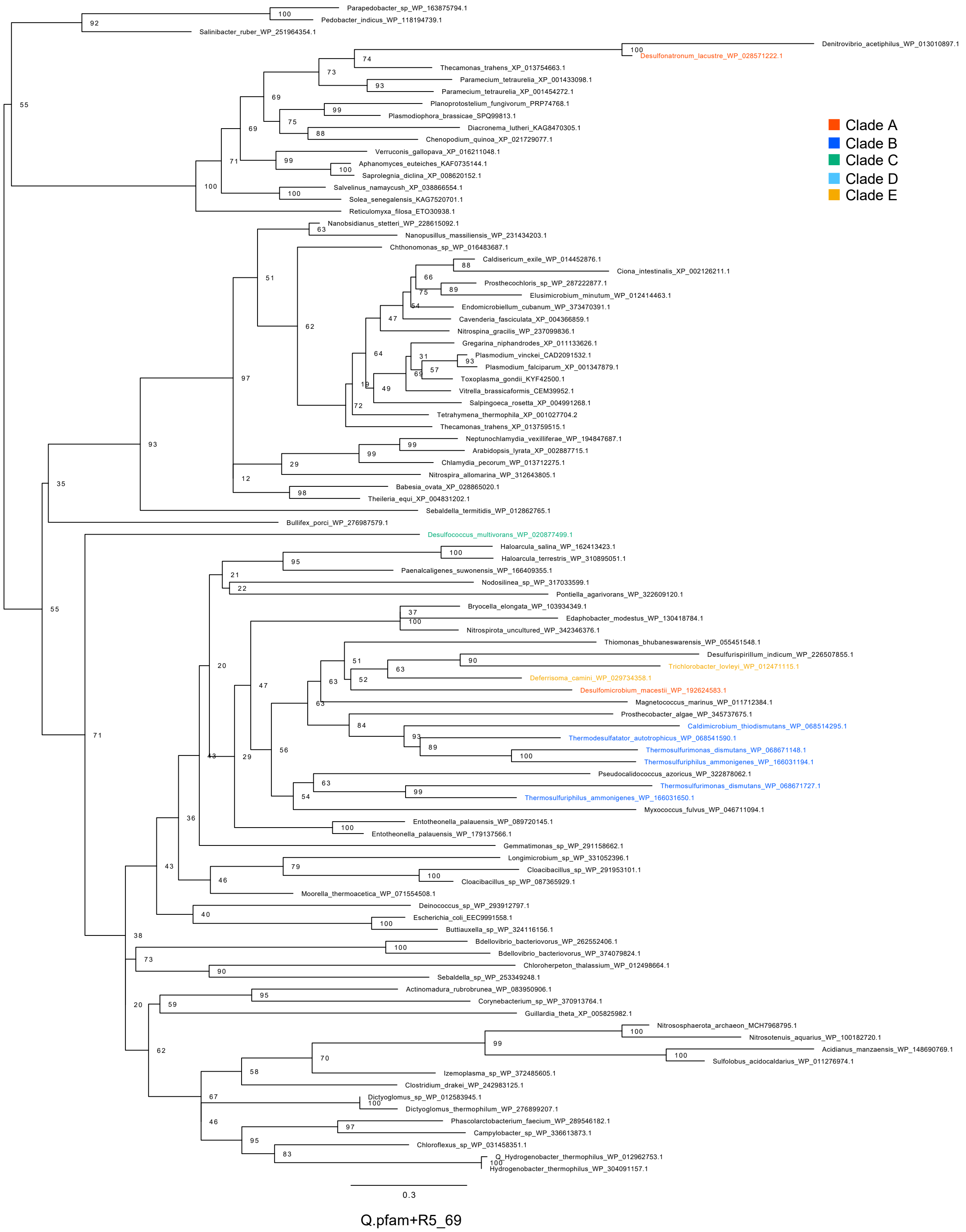

Supplementary Figure 6. Maximum likelihood phylogenetic tree of PSP
(A) dPSP1; (B) dPSP2; (C) iPSP1. Bootstrap values were calculated using the ultrafast bootstrap method. The evolutionary model, scale bar and number of sites are shown in the figure. Sequences in the phylum Desulfobacterota are highlighted with colors.

【Fig. S7. Maximum likelihood phylogenetic tree of SHMT.】

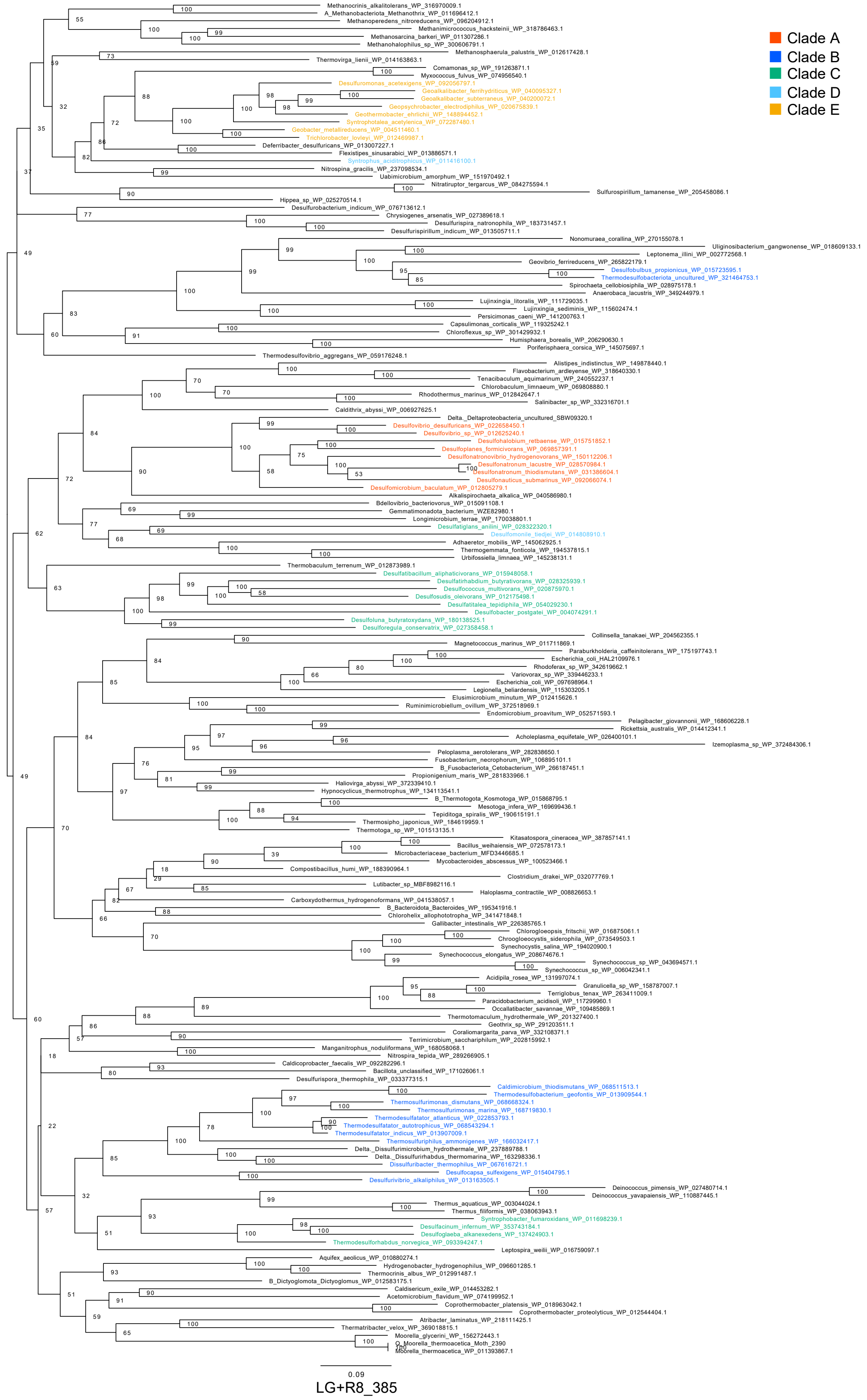

Supplementary Figure 7. Maximum likelihood phylogenetic tree of SHMT.
Bootstrap values were calculated using the ultrafast bootstrap method. The evolutionary model, scale bar and number of sites are shown in the figure. Sequences in the phylum Desulfobacterota are highlighted with colors.

【 Fig. S8A. Maximum likelihood phylogenetic tree of GCSP β-subunit.】

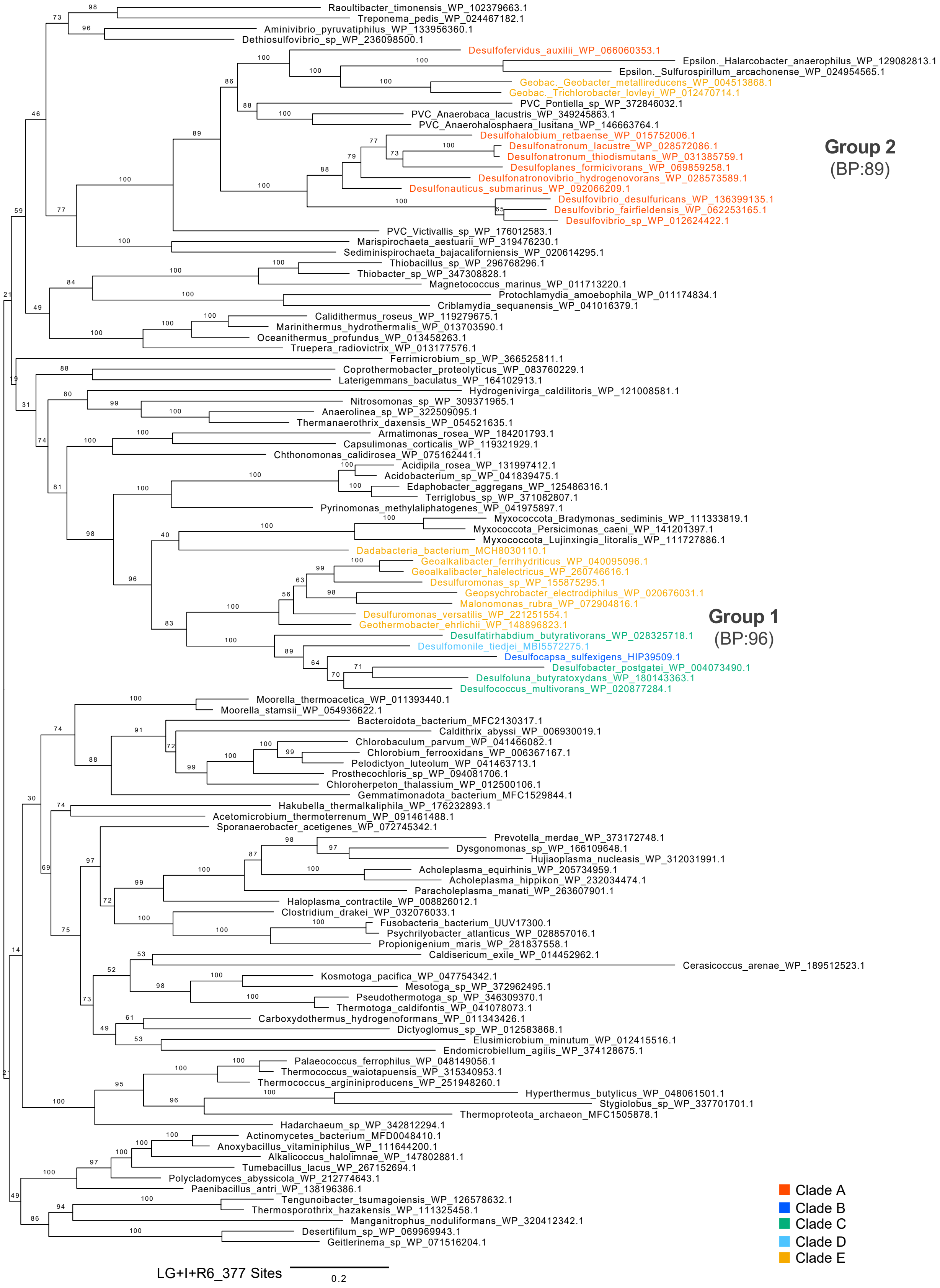

【 Fig. S8B. Maximum likelihood phylogenetic tree of GCST.】

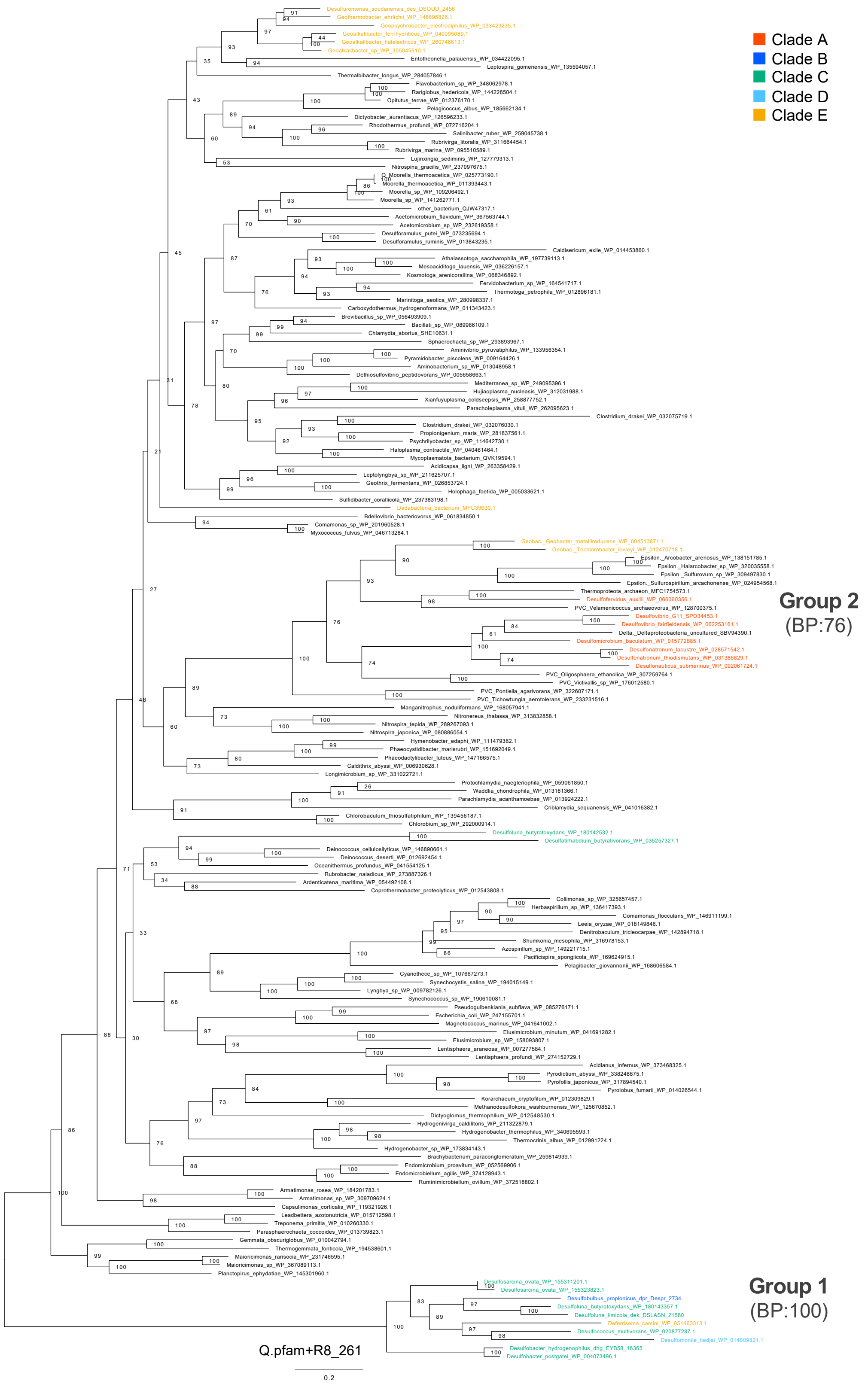

【 Fig. S8C. Maximum likelihood phylogenetic tree of GCSL.】

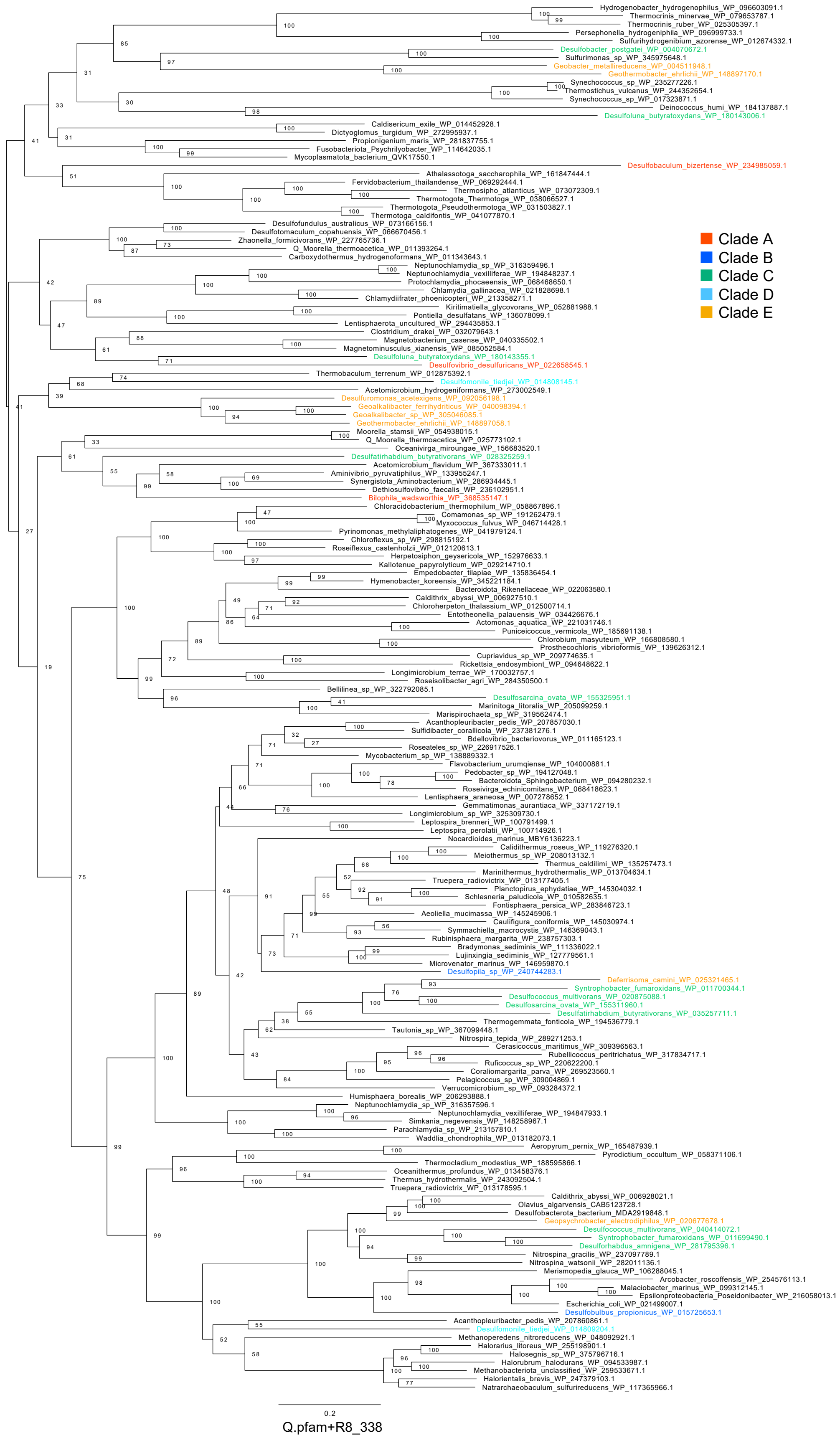

【 Fig. S8D. Maximum likelihood phylogenetic tree of GCSH.】

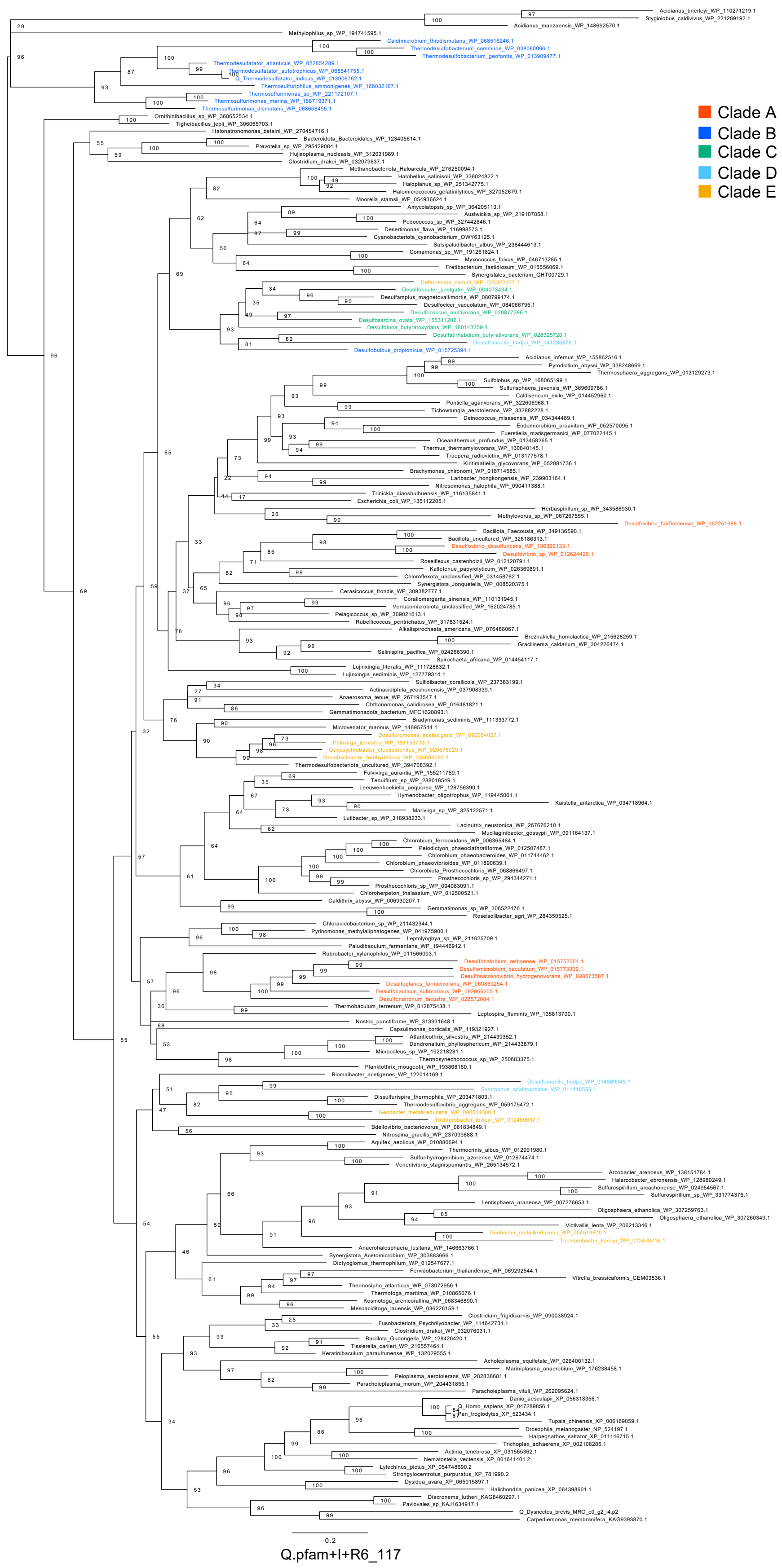

Supplementary Figure 8. Maximum likelihood phylogenetic tree of the GCS complex.
(A) GCSP β-subunit; (B) GCST; (C) GCSL; (D) GCSH. Bootstrap values were calculated using the ultrafast bootstrap method. The evolutionary model, scale bar and number of sites are shown in the figure. Sequences in the phylum Desulfobacterota are highlighted with colors.

【Fig. S9A. Maximum likelihood phylogenetic tree of GR-A.】

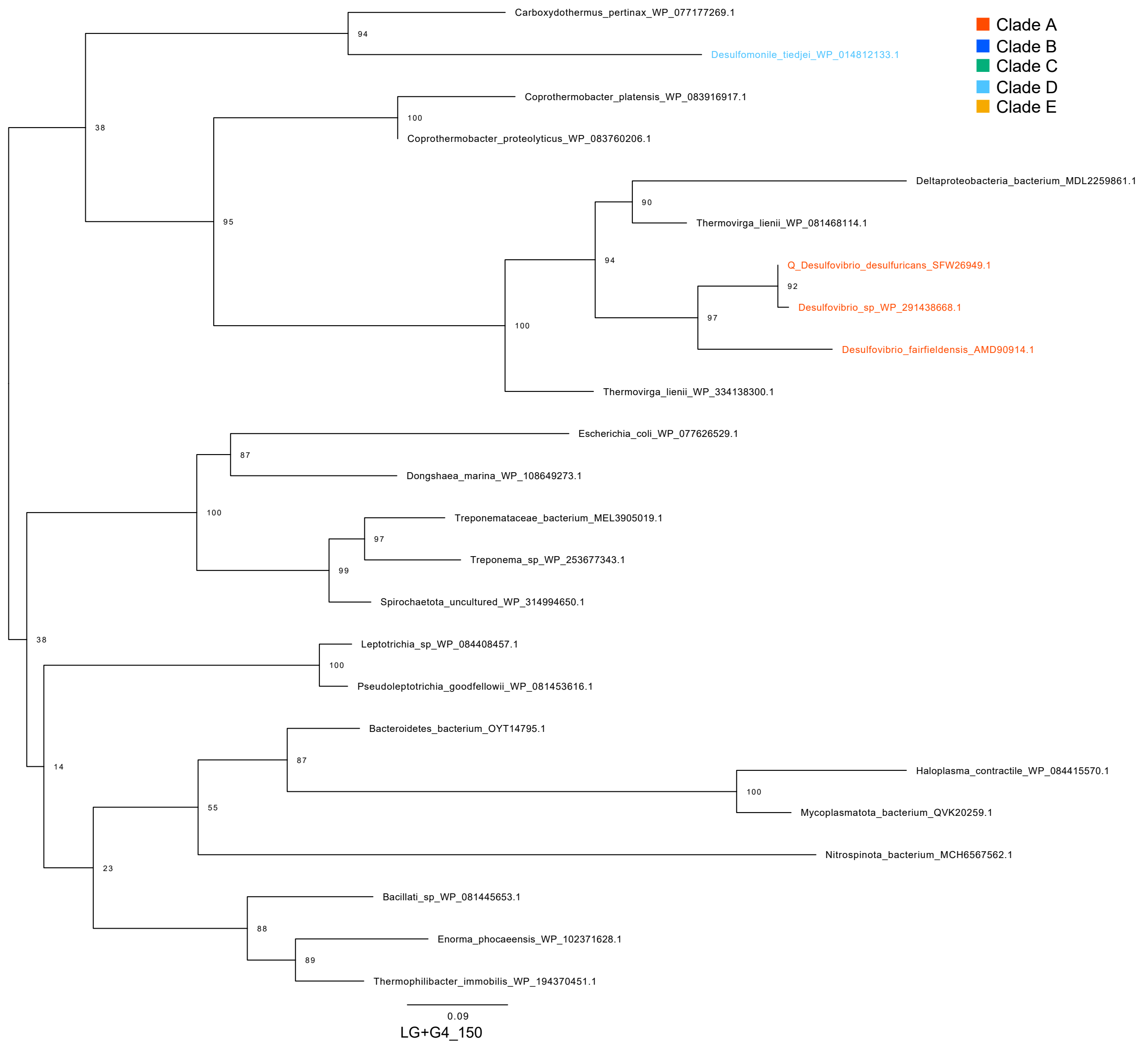

【 Fig. S9B. Maximum likelihood phylogenetic tree of GR-Bab.】

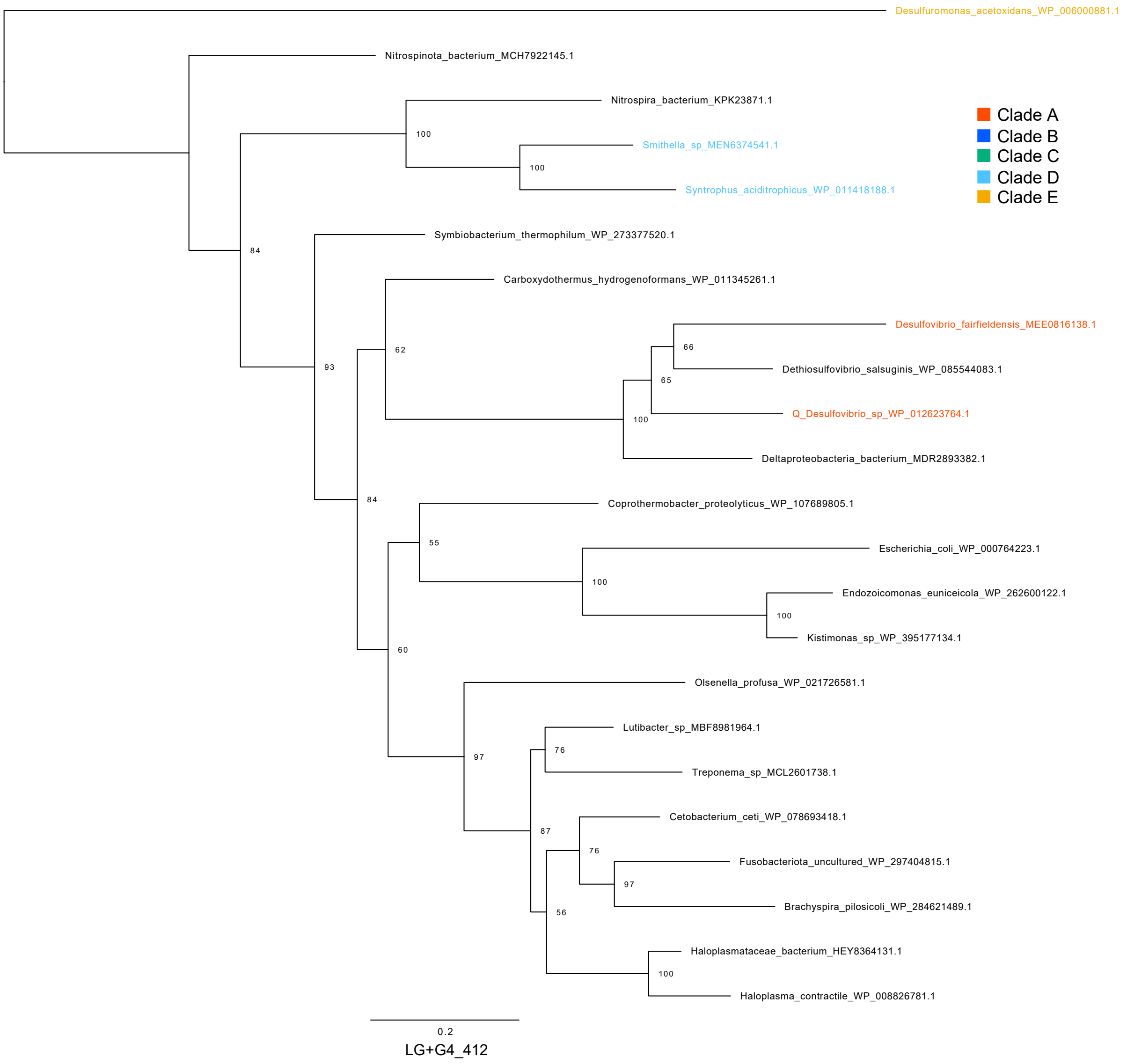

【 Fig. S9C. Maximum likelihood phylogenetic tree of GR-Bg.】

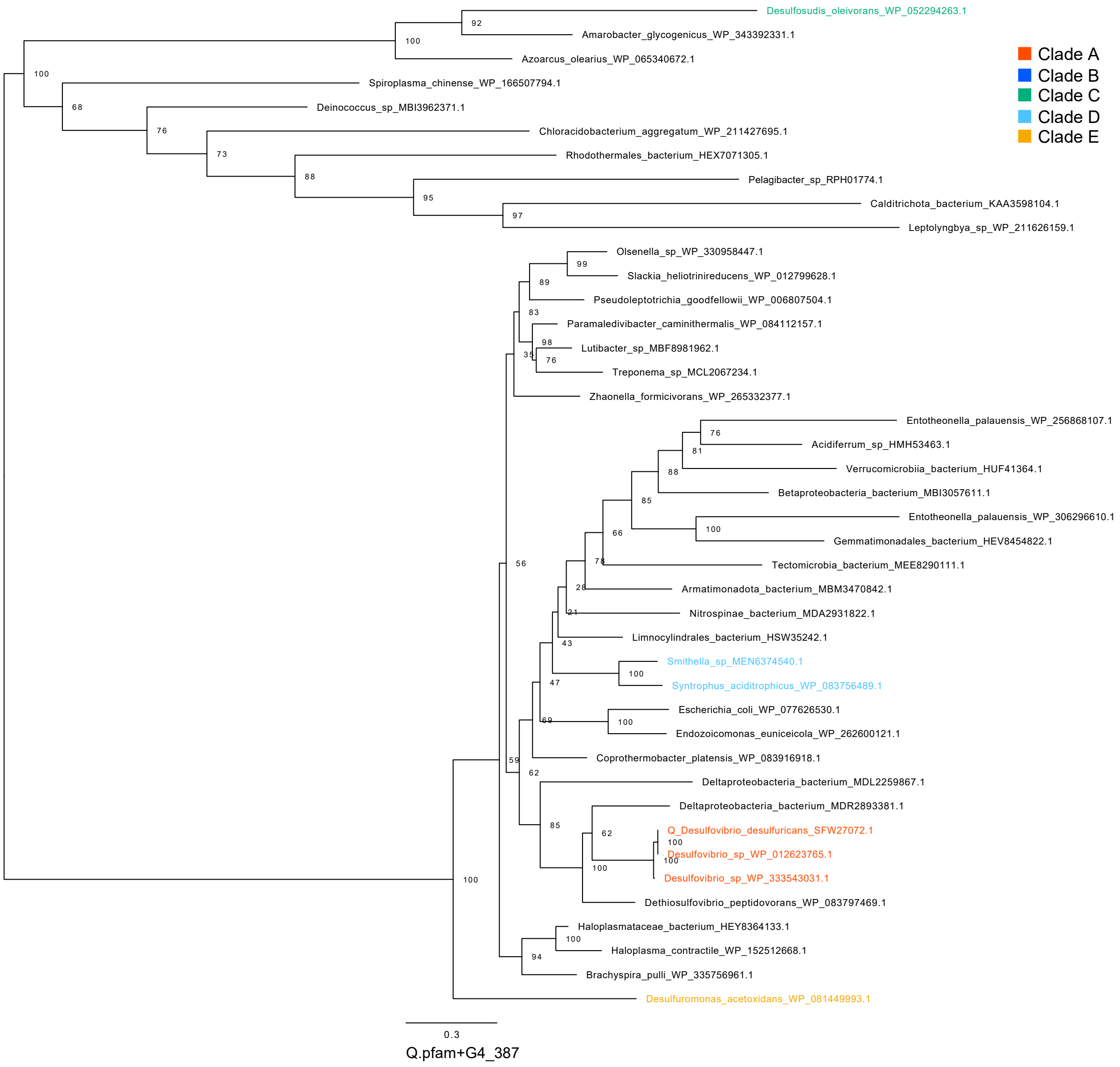

【Fig. S9D. Maximum likelihood phylogenetic tree of GR-Ca.】

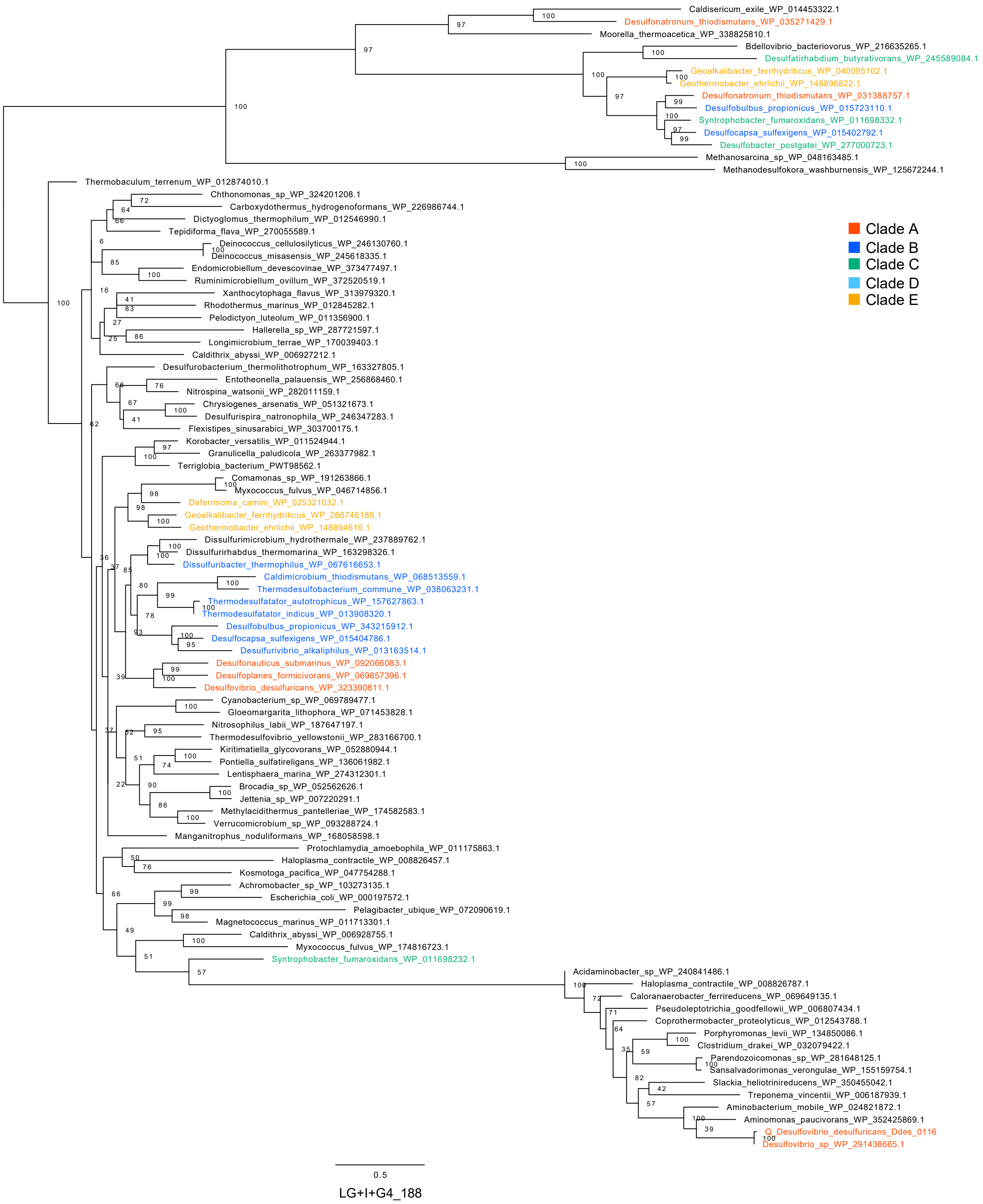

)

【Fig. S9E. Maximum likelihood phylogenetic tree of GR-Cb.】

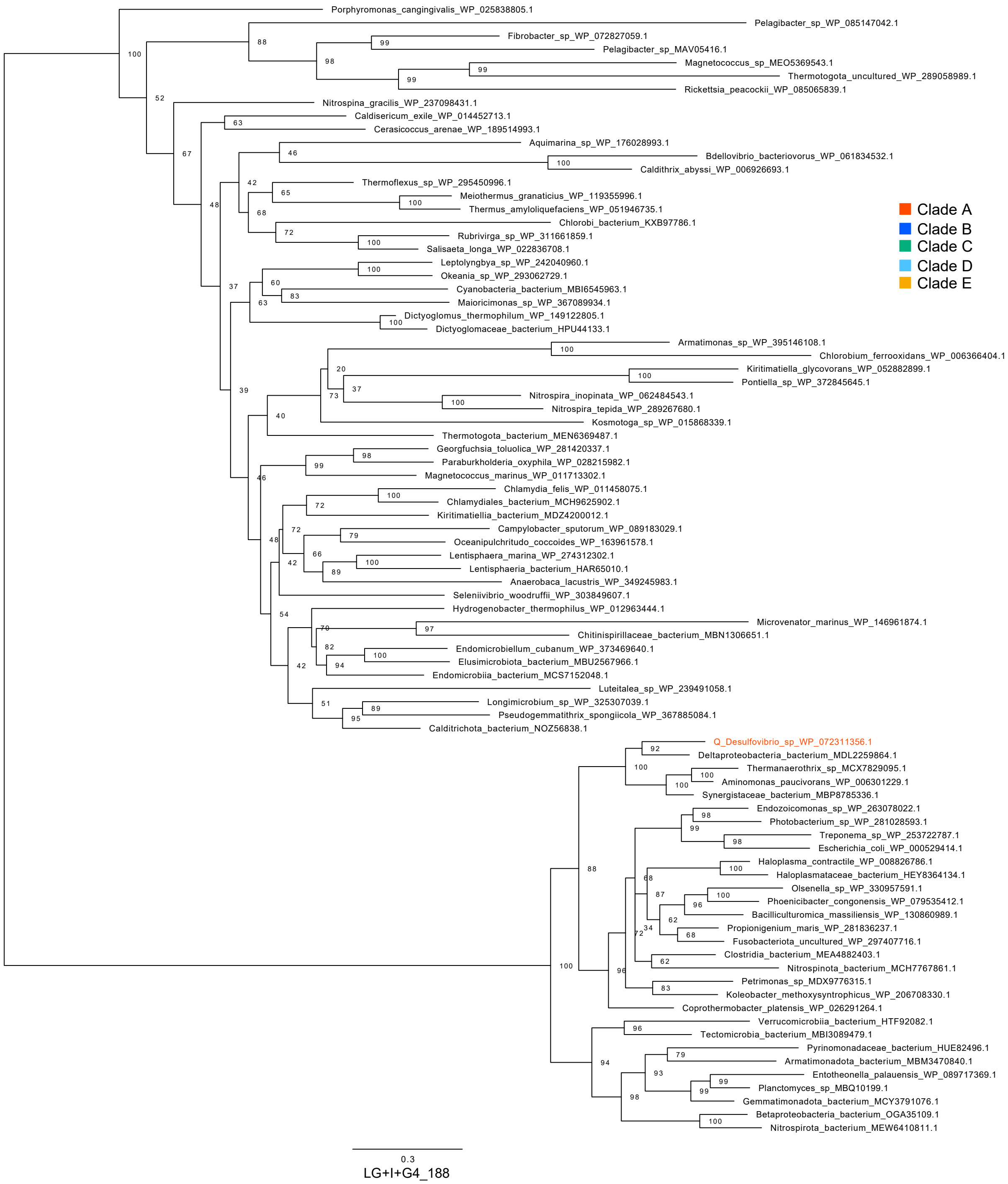

Supplementary Figure 9. Maximum likelihood phylogenetic tree of the GR.

(A) GR-A; (B) GR-Bab; (C) GR-Bg; (D) GR-Ca; (E) GR-Cb. Bootstrap values were calculated using the ultrafast bootstrap method. The evolutionary model, scale bar and number of sites are shown in the figure. Sequences in the phylum Desulfobacterota are highlighted with colors.

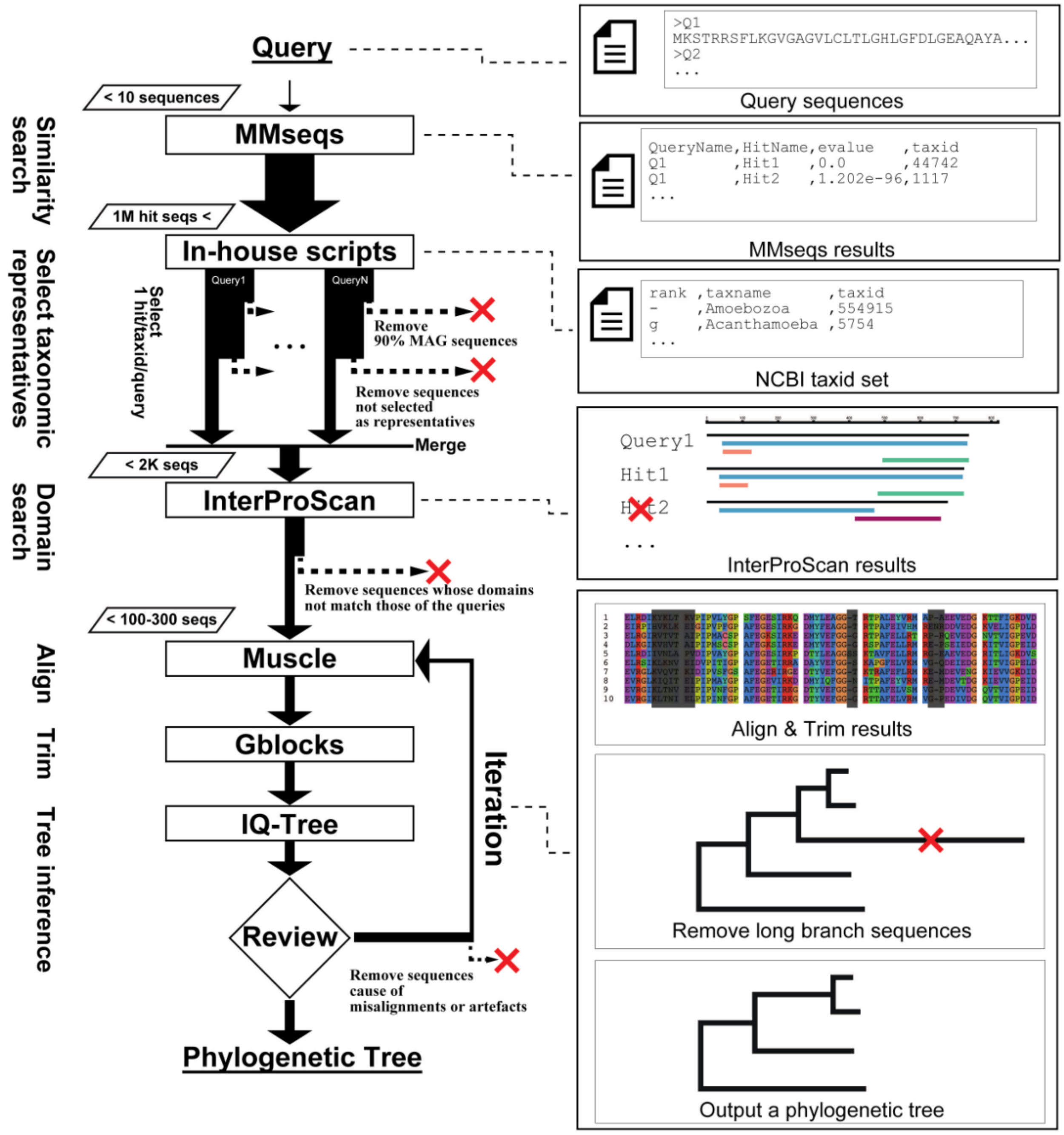

Supplementary Figure 10. Workflows for phylogenetic analysis.

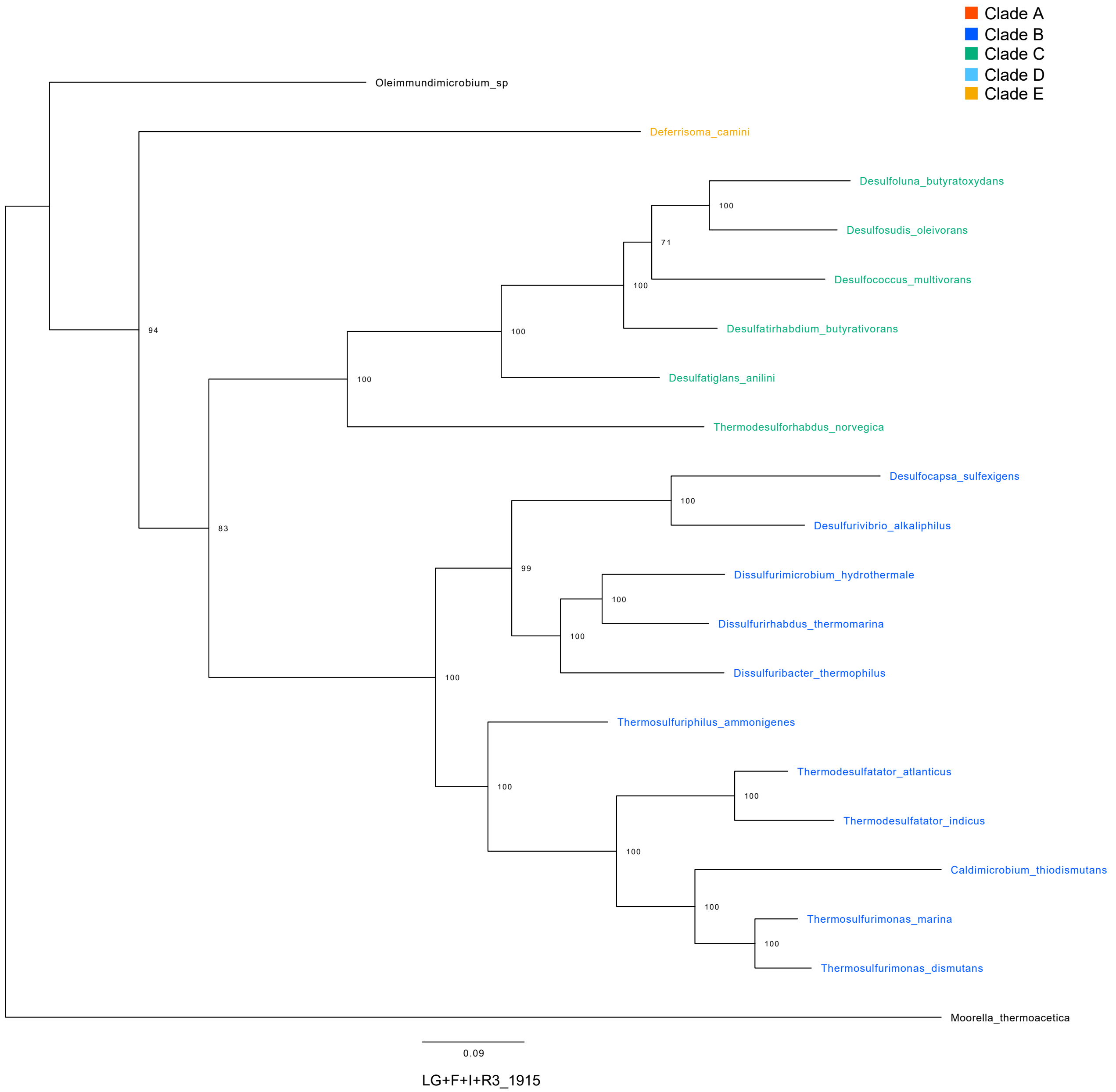

Supplementary Figure 11. Concatenated phylogenetic tree of CODH/ACS complex. Maximum likelihood phylogenetic tree constructed from concatenated protein sequences of AcsA to E subunits. Bootstrap values were calculated using the ultrafast bootstrap method. The evolutionary model, scale bar and number of sites are shown in the figure. Sequences in the phylum Desulfobacterota are highlighted with colors.
